# Design and Application of Stimuli-Responsive Hydrogels for 4D Printing: A Review of Adaptive Materials in Engineering

**DOI:** 10.3390/gels12020138

**Published:** 2026-02-02

**Authors:** Muhammad F. Siddique, Farag K. Omar, Ali H. Al-Marzouqi

**Affiliations:** 1 Mechanical and Aerospace Engineering Department, College of Engineering, United Arab Emirates University, Al Ain 15551, United Arab Emirates; 700042442@uaeu.ac.ae; 2 Chemical and Petroleum Engineering Department, College of Engineering, United Arab Emirates University, Al Ain 15551, United Arab Emirates; hassana@uaeu.ac.ae

**Keywords:** stimuli-responsive hydrogels, 4D printing, adaptive materials, shape-morphing systems, hydrogel actuators, functional material design, advanced manufacturing

## Abstract

Stimuli-responsive hydrogels are an emerging class of smart materials with immense potential across biomedical engineering, soft robotics, environmental systems, and advanced manufacturing. In this review, we present an in-depth exploration of their material design, classification, fabrication strategies, and real-world applications. We examine how a wide range of external stimuli—such as temperature, pH, moisture, ions, electricity, magnetism, redox conditions, and light—interact with polymer composition and crosslinking chemistry to shape the responsive behavior of hydrogels. Special attention is given to the growing field of 4D printing, where time-dependent shape and property changes enable dynamic, programmable systems. Unlike existing reviews that often treat materials, stimuli, or applications in isolation, this work introduces a multidimensional comparative framework that connects stimulus-response behavior with fabrication techniques and end-use domains. We also highlight key challenges that limit practical deployment—including mechanical fragility, slow actuation, and scale-up difficulties—and outline engineering solutions such as hybrid material design, anisotropic structuring, and multi-stimuli integration. Our aim is to offer a forward-looking perspective that bridges material innovation with functional design, serving as a resource for researchers and engineers working to develop next-generation adaptive systems.

## 1. Introduction

Hydrogels are water-rich polymer networks whose mechanical properties, swelling behavior, and transport characteristics can be precisely engineered through polymer chemistry and crosslinking design. When integrated with additive manufacturing, these features enable the fabrication of structures that can deform, reconfigure, or adapt their properties over time in response to external stimuli [1,2]. Hydrogels can be produced from nearly any water-soluble polymer, encompassing a broad spectrum of chemical compositions and bulk physical properties. Moreover, hydrogels can be designed in various physical forms, including slabs, microparticles, nanoparticles, coatings, and films [3].

**Figure 1:**
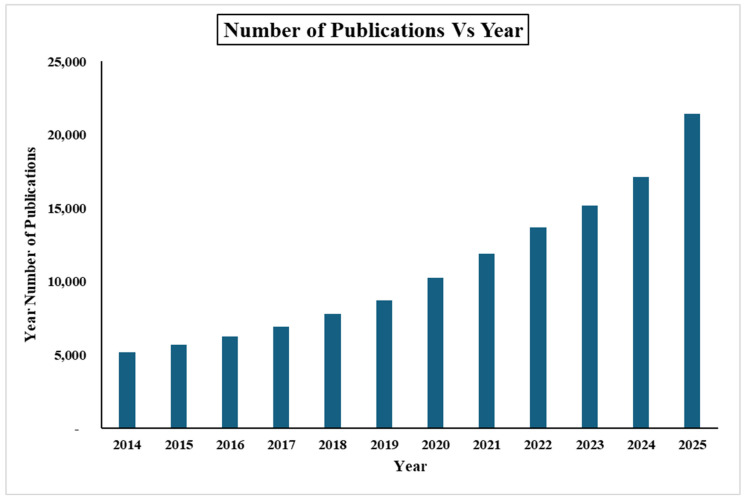
Number of hydrogel publications (source: Scopus; keyword: Hydrogel; December 2025).

In recent decades, advanced hydrogel materials have garnered significant research interest [4]. Figure 1 shows the number of publications on the topic of hydrogels in the last decade. Due to their tunable properties and functionalities, along with their simple preparation methods, hydrogels play a vital role in various biomedical and engineering applications. These applications include tissue-engineering scaffolds, drug-delivery systems, soft contact lenses, antifouling coatings, sensors, actuators, soft robotics, and wastewater treatment [5,6,7,8,9,10,11,12].

**Figure 2:**
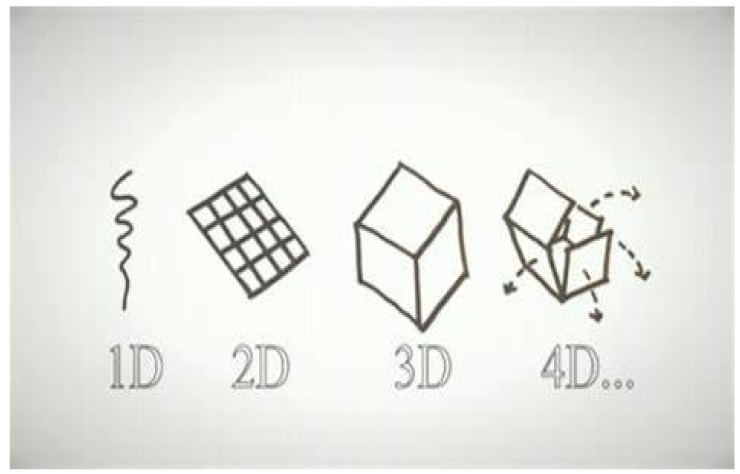
Illustration comparing 1D, 2D, and 3D static forms with 4D structures, where time acts as an active dimension, allowing printed objects to autonomously deform, reconfigure, or adapt their properties in response to environmental stimuli [20].

Recently emerging from 3D printing technology, 4D printing showcases significant potential and promising capabilities for a wide range of applications [13]. 4D printing was initiated and coined by a research group at Massachusetts Institute of Technology [14]. It leverages the rapid advancements in smart materials, 3D printing technology, and mathematical modeling and design [15]. 4D printing was originally defined as “4D printing = 3D printing + time,” emphasizing the added dimension of time, where printed structures can change shape or function over time in response to external stimuli. Figure 2 provides a basic illustration of the 4D printing concept, showing how the shape, properties, or functionality of a 3D-printed structure can evolve over time [16,17,18]. 4D printing represents a deliberate advancement of the 3D printed structure, encompassing changes in shape, properties, and functionalities. It facilitates self-assembly, multifunctionality, and self-repair capabilities. Additionally, it is characterized by its time-dependence, independence from specific printers, and predictability. Unlike traditional 3D-printed devices, 4D-printed devices can interact with their environment by responding to external stimuli, generating various outputs such as mechanical movements or biological responses. As a result, 4D printing techniques have attracted significant attention in both academic and industrial fields [19].

The emergence of hydrogels through 4D printing has captured the attention of scholars and scientists, leading to a wave of discoveries and advancements in this innovative area [21,22,23,24,25]. The convergence of hydrogel innovation with 4D printing not only highlights the interdisciplinary partnerships propelling the next wave of smart technology but also the swift advancement of material science. Hydrogels in 4D printing have enormous promise and might have revolutionary effects on both business and daily life as research into the material advances.

Several excellent reviews have already surveyed stimuli-responsive hydrogels and hydrogel-based additive manufacturing from different perspectives. Koetting et al. and Ullah et al. [26,27] provided broad overviews of stimulus-responsive hydrogels, emphasizing fundamental theory, classification, and biomedical applications. Sun et al. and Hendi et al. [28,29] focused on specific sensing and healthcare-related implementations, while Nasution et al. [30] in Gels discussed cellulose-based hydrogels with an emphasis on crosslinking effects. Champeau et al. [31] reviewed 4D printing of hydrogels from a process and materials perspective. More recently, dedicated reviews appeared on Gels related to stimuli-responsive hydrogels and their applications, on smart drug-delivery carriers based on stimuli-responsive hydrogels and nanogels, and on 3D-printed hydrogels for diverse applications, which together consolidate the state of the art in hydrogel chemistry, stimuli-driven functions, and printing technologies [32,33,34].

Existing classification schemes for stimuli-responsive hydrogels typically organize materials along a single dominant axis, such as stimulus type, polymer chemistry, crosslinking mechanism, or application domain. Existing single-axis classification approaches are effective for material cataloguing but provide limited insight into how molecular design, printed architecture, and fabrication strategy collectively govern time-dependent deformation, mechanical evolution, and functional reliability in adaptive systems [31,35,36]. The rationale for adopting a multidimensional framework stems from the fact that 4D-printed hydrogel systems operate as joint material–structure–stimulus systems, where functional performance emerges from interactions across multiple design layers rather than from any single parameter alone. While existing approaches are valuable for cataloguing materials, they often treat fabrication strategies, structural architecture, and functional outcomes as secondary considerations and therefore provide limited guidance for the design of adaptive, 4D-printed systems [36,37]. By explicitly integrating stimulus mechanisms, hydrogel network design, and additive manufacturing strategies, the proposed framework enables a system-level understanding that links material formulation to printed structure and dynamic response, thereby supporting rational design and programming of 4D-printed hydrogel systems beyond the scope of conventional review approaches [38,39,40]. In contrast to regenerative medicine-oriented reviews that primarily emphasize biological performance, cell–material interactions, and static scaffold functionality [41,42,43], the present work adopts an engineering-driven perspective focused on programmable, time-dependent behavior in 4D-printed hydrogel systems, where functional performance emerges from the coupled design of material chemistry, printed architecture, and stimulus activation.

In contrast to these earlier works, this review specifically targets stimuli-responsive hydrogels for 4D printing and adaptive engineering systems. We introduce a multidimensional classification framework that simultaneously links (i) stimulus type (thermal, pH, moisture, ionic, electric, magnetic, redox, and light), (ii) hydrogel composition and crosslinking chemistry, and (iii) fabrication strategies, with particular attention to 3D/4D printing methods and printed architectures. By integrating this framework with an extensive comparative table that maps materials, fabrication techniques, and end-use domains, we move from a purely material- or application-centric perspective to a design-oriented, system-level view. Furthermore, we synthesize engineering guidelines to tackle practical bottlenecks such as mechanical fragility, slow actuation, environmental stability, and scale-up, thereby positioning this work as a bridge between stimuli-responsive hydrogel science and functional device engineering in biomedical, soft robotic, environmental, and energy-related applications.

## 2. Classification of Hydrogels

**Figure 3:**
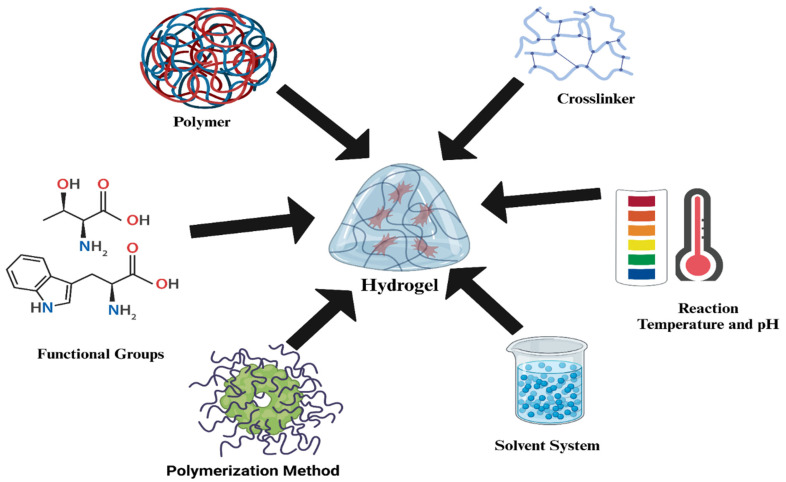
Key parameters influencing hydrogel synthesis.

**Figure 4:**
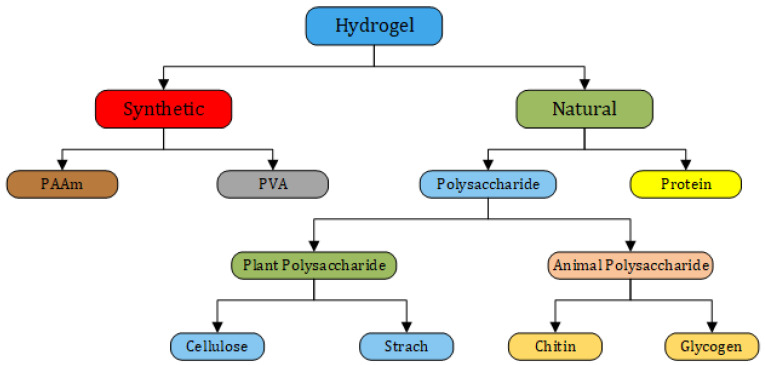
Types of materials used for hydrogel synthesis based on their origin.

Hydrogels can be classified in various ways. However, since hydrogels are fundamentally composed of cross-linking networks, they can be categorized based on their cross-linking methods into two main types: (a) physically cross-linked or self-assembled hydrogels, and (b) chemically cross-linked hydrogels [44,45]. Several types of chemical and physical hydrogels have been developed using natural and synthetic polymers, and they have been utilized in a wide range of applications. Physical or chemical interactions or functional groups involved in the synthesis enhance the hydrogel structure, creating a new compound tailored for specific applications. The key components necessary for hydrogel production are illustrated in Figure 3. Figure 4 represents the types of materials used for hydrogel synthesis based on their origin.

## 3. Stimuli-Responsive Hydrogels

**Figure 5:**
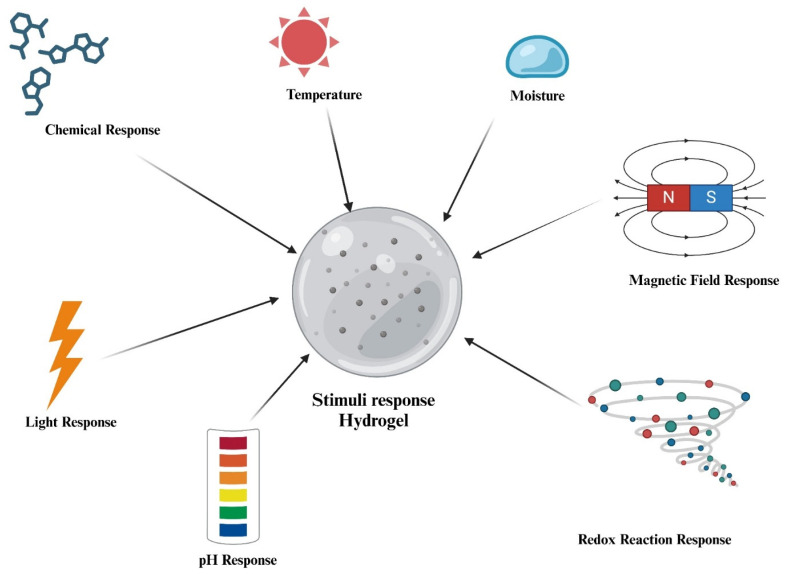
Multifunctional stimuli-responsive hydrogel: A schematic overview of key response mechanism.

Stimuli-responsive hydrogels are a diverse group of hydrogels that can undergo switchable transitions from gel to solution or gel to solid in response to external stimuli. External stimuli such as temperature, moisture, pH, ionic strength, electric fields, magnetic fields, redox reactions, and light can trigger deformation in printed structures by altering polymer–solvent interactions or crosslinking dynamics. The effectiveness of these responses is strongly influenced by printed feature size, network elasticity, and stimulus penetration depth [29,46,47]. Figure 5 represents an illustration of responses that can generate stimuli in smart hydrogels.

Unlike conventional hydrogel applications that prioritize static swelling behavior, 4D-printed hydrogel systems require predictable, repeatable, and spatially controllable responses [48]. As the movement of ions, molecules, and water into the hydrogel primarily occurs through diffusion, the time required for volumetric changes is influenced by the diffusion distance or the size of the hydrogels [49]. Scaling down the size of hydrogel structures to micrometers can result in response times ranging from a few seconds to sub-seconds [50]. Shape-changing hydrogels that are most relevant for 4D printing deliberately couple a well-defined stimulus (thermal, optical, magnetic, hydraulic/pneumatic, or ionic) to programmed architectures that convert local swelling/shrinkage or stiffness changes into predictable motion. A recent study [51] demonstrates these approaches across length scales: two-photon polymerization of Poly(N-isopropylacrylamide) (PNIPAM) microarchitectures shows reversible, temperature-driven micro-morphing for microscale devices. At the mesoscale, femtosecond-laser 4D printing achieves precise photothermal/optical actuation in smart hydrogel microstructures, enabling rapid, repeatable deformation with high spatial control [52]. New Digital Light Processing (DLP)/vat-photopolymerization strategies produce tough, multi-responsive hydrogel constructs that combine photopolymerizable networks with nanofillers to improve recovery force and durability for soft actuators [53]. Structural–topology approaches that exploit filament spacing and gradient architectures have been used to program ion-induced swelling and Ca^2+^-triggered morphing, highlighting that design (topology + material) rather than chemistry alone governs many practical 4D outcomes [54]. Finally, high-swelling composite hydrogels (e.g., Gelatin Methacryloyl (GelMA) + superabsorbent components) have been demonstrated to deliver large, controllable volumetric changes suitable for actuator and soft-robotic functions when combined with appropriate printing modalities [55].

The performance of stimuli-responsive hydrogels is not determined by environmental stimuli alone but rather emerges from the coupled interaction between external triggers and molecular design parameters within the hydrogel network [31,39,40]. For a given stimulus, such as temperature, pH, or ionic strength, the magnitude, rate, and reversibility of the hydrogel response are strongly governed by polymer composition, crosslinking density, network architecture, and the presence of functional or reinforcing components [32,35].

Single-stimulus hydrogel systems often suffer from limited robustness and scalability due to narrow operating windows and strong sensitivity to environmental fluctuations. In contrast, multi-stimuli-responsive hydrogels enhance functional robustness by providing complementary or redundant activation pathways, allowing consistent actuation or property modulation even when one stimulus becomes ineffective [38,56]. From a scalability perspective, integrating multiple stimuli (e.g., thermal–ionic, optical–magnetic, or electro–magnetic) reduces dependence on precise environmental control and enables remote, hierarchical, or sequential activation across larger printed structures, which is difficult to achieve using single-stimulus systems alone [38,57]. Consequently, multi-stimuli integration improves tolerance to environmental variability, operational reliability, and manufacturability, making such systems more suitable for scalable and real-world 4D-printed applications [38,40].

In the following subsections, stimuli-responsive hydrogels are discussed from a 4D-printing perspective, emphasizing how different stimuli influence actuation mechanisms, response speed, and structural reliability in printed systems. Furthermore, we will delve into the limitations and strong pursuits of each response. To give the reader an overview of the recent publications, Table 1 represents commonly reported stimuli-responsive hydrogel matrices including various types that respond to different stimuli, fabrication techniques, and applications.

### 3.1. Moisture-Responsive Hydrogels

**Figure 6:**
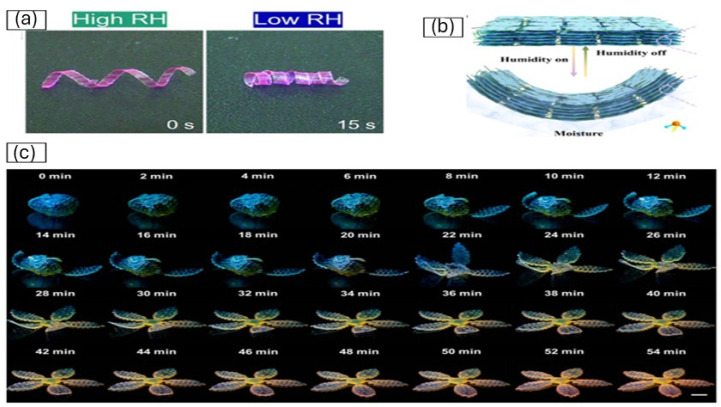
The programmable morphing of the YYI actuator under different humidity levels. (**a**) A left-handed coil actuator with the PAM hydrogel on the inside under high relative humidity (≈60–75% RH) conditions [59]; (**b**) Time-lapse images of the biomimetic anthesis process of the initially fully swelled printed hydrogel flower at a relative humidity condition of 20%, exhibiting simultaneous blue-to-red luminescence color tuning and shape morphing [60]; (**c**) Schematic diagram illustrating the actuation mechanism of the humidity-responsive actuator for the CNF/GO/CNT composite film [68].

Moisture-driven shape morphing in 4D-printed systems is often achieved through asymmetric swelling designs [58]. Jingjing Li et al. [59] demonstrated a Yin–Yang interface (YYI) actuator by coupling a moisture-responsive polyacrylamide hydrogel layer with a moisture-inert PET layer, where differential swelling under humid conditions induced predictable bending. Figure 6a represents the controllable morphing of the YYI actuator in response to varying humidity levels. Yuan Yao et al. [60] reported a 3D-printable zwitterionic supramolecular hydrogel that exhibited humidity-triggered deformation accompanied by tunable luminescence, enabling multifunctional actuation. Figure 6b demonstrates time-lapse images of the biomimetic anthesis process of the initially fully swelled printed hydrogel flower. In another study related to actuators, Yifan Zheng et al. [61] reported a 3D-printable zwitterionic supramolecular hydrogel that exhibited humidity-triggered deformation accompanied by tunable luminescence, enabling multifunctional actuation.

A conductive hydrogel is a semi-solid, three-dimensional interconnected porous network that features adjustable chemical and physical interactions along with excellent conductivity [8,62]. Zhenzhen Liu et al. [63] developed highly compressible conductive hydrogel sensors that exhibit synergistic properties, including long-lasting moisture retention, extreme temperature tolerance, and strain sensitivity. In this study, a dual-crosslinked hydrogel electrolyte was developed by immersing a photo-crosslinked polyvinyl alcohol (PVA-W) hydrogel in a solvent containing tannic acid, NaCl, glycerin, and water. However, their diffusion-limited response speed and mechanical weakness in highly swollen states can restrict practical applications.

Water evaporation and freezing limit the durability of hydrogel-based 4D-printed structures [64]. Yinjie Peng et al. [65] improved environmental tolerance by incorporating glycerin into a Poly(3,4-ethylenedioxythiophene)/Polyvinyl Alcohol (PVA/PEDOT) hydrogel, while the addition of Poly(acrylic acid) (PAA) restored mechanical strength, enabling stable actuation under low-temperature and low-humidity conditions.

Cellulose-based hydrogels have gained attention for moisture-responsive 4D applications due to their sustainability and strong hydrophilicity [66]. A study [67] details the synthesis and thorough characterization of water-responsive hybrid hydrogels composed of Carboxymethyl Cellulose (CMC) and Polyvinyl Alcohol (PVA), using citric acid as the crosslinking agent. Hybrid CMC/PVA hydrogels crosslinked with citric acid demonstrate tunable swelling behavior through control of composition and crosslink density, enabling programmable moisture-driven deformation. In addition, cellulose nanofiber (CNF)-based composite films incorporating graphene oxide (GO) and carbon nanotubes (CNT) exhibit enhanced humidity-induced actuation, where improved water uptake and transport translate into reliable shape change, highlighting the potential of cellulose-derived systems for moisture-responsive 4D-printed structures. Figure 6c represents the deformation and underlying mechanisms of the CNF/GO/CNT composite film.

Comparative analysis of moisture-responsive hydrogels indicates that bilayer and hybrid systems outperform monolithic hydrogels by providing more predictable bending and reduced mechanical fatigue. Designs that decouple the swelling layer from a mechanically reinforcing layer achieve higher actuation reliability, demonstrating that architectural asymmetry enabled by printing is more important than swelling capacity alone.

### 3.2. Thermo-Responsive Hydrogels

Thermo-responsive hydrogels undergo reversible volume or phase changes in response to temperature variations and are among the most widely used materials in 4D printing due to the ease and precision of thermal control [69]. Thermosensitive hydrogels exhibit unique behavior: they form a gel at higher temperatures and revert to a liquid state at lower temperatures within a specific temperature range. This behavior is contrary to the conventional melt transition behavior typically observed in many materials [70].

PNIPAm is the most widely used thermo-responsive polymer in 4D printing due to its sharp and reversible volume transition near physiological temperature (Lower Critical Solution Temperature (LCST) ≈ 32 °C), which enables predictable temperature-driven shape transformation [71]. In thermo-responsive hydrogels based on PNIPAM, the LCST, actuation strain, and response kinetics are not intrinsic material constants but can be systematically tuned through copolymerization, crosslinker type and concentration, and network topology [72,73,74]. In printed systems, PNIPAM-based hydrogels contract above the LCST and swell below it, providing a reliable mechanism for thermally programmed actuation [75]. Among thermo-responsive homopolymers, PNIPAm has a well-characterized structure, and its LCST is 32 °C [76,77,78]. Tobias Spratte et al. [74] fabricated PNIPAM microactuators via direct laser writing, enabling precise control of printed geometry and reproducible temperature-induced swelling and shrinkage behavior. This process involves the polymerization of a photo-sensitive resistive hydrogel composed of NIPAM monomers, MBA comonomers, and lithium phenyl-2,4,6-trimethylbenzoylphosphinate (LAP) as a PI via two-photon absorption. However, single-network PNIPAM systems are often mechanically limited in bulk printed structures. However, to address this, Lukas Bauman et al. [73] developed a composite double-network PNIPAM hydrogel using VAT polymerization, in which the incorporation of a secondary thermo-responsive network enhanced mechanical stability and enabled multi-stage thermal response through the emergence of two distinct LCSTs. Notably, the hydrogel demonstrated significant variations in strength as it collapsed, aligning with the expected behavior of PNIPAm-based hydrogels.

**Figure 7:**
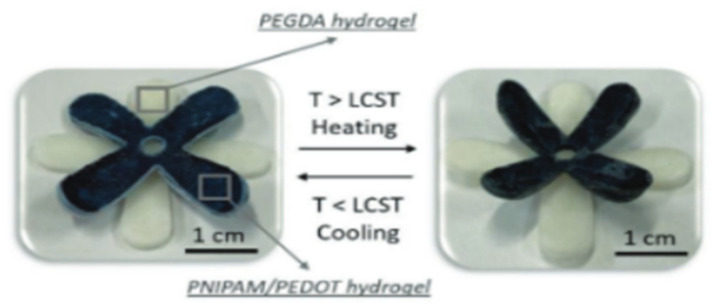
Stimuli-responsiveness of 3D-printed PNIPAM/PEDOT (1.3 wt.%) hydrogel and PEGDA hydrogel in lotus blossom shape below and above LCST (35 °C) [79].

Beyond mechanical actuation, PNIPAM has also been integrated into multifunctional 4D-printed devices. Naroa Lopez-Larrea et al. [79] combined PNIPAM with PEDOT:PSS to create thermo-responsive conductive hydrogels, where temperature-driven volume changes were coupled with pronounced variations in mechanical and electrical properties, demonstrating the potential of PNIPAM-based systems for adaptive bioelectronic and sensing applications in 4D printing. Figure 7 represents the stimuli-responsiveness of 3D-Printed Poly(3,4-ethylenedioxythiophene):Poly(styrenesulfonate) (PNIPAM/PEDOT) (1.3 wt.%) hydrogel and PEGDA in a lotus blossom shape below and above LCST (35 °C).

Beyond actuation and drug delivery, PNIPAM-based hydrogels have also been explored for thermo-responsive water treatment applications relevant to 4D-printed systems. Zhang et al. [80] developed an ionic PNIPAM/γ-PGA/PEG hydrogel drawing agent for forward-osmosis desalination, demonstrating temperature-controlled water flux and good recyclability. Similarly, Yunsong Liu et al. [81] reported a thermo-responsive hydrogel evaporator fabricated via UV-cured 3D printing, where temperature-induced phase transitions enhanced water transport efficiency and structural durability for solar steam generation.

These examples demonstrate that PNIPAM-based hydrogels enable functional expansion of 4D-printed systems beyond mechanical actuation into adaptive water treatment technologies. More broadly, PNIPAM-based hydrogels dominate thermo-responsive 4D-printing applications due to their sharp LCST near physiological temperature and compatibility with photopolymerization-based printing methods. However, single-network PNIPAM systems consistently underperform in mechanical durability, whereas double-network and composite PNIPAM hydrogels exhibit superior shape retention and cyclic stability. This comparison highlights that network architecture, rather than polymer chemistry alone, governs long-term performance in thermo-responsive 4D-printed systems [82].

### 3.3. Chemical-Responsive Hydrogel

Chemical-responsive hydrogels exhibit stimulus-induced deformation in response to environmental parameters such as pH and ionic strength, making them widely applicable in 4D-printed sensors, actuators, and soft robotic systems [83]. In these materials, stimulus-induced deformation arises from reversible ionization, crosslinking, or dissociation of polymer chains upon interaction with surrounding ions, leading to controlled volumetric changes in printed architectures [84,85]. Among chemical-responsive systems, pH-responsive hydrogels are particularly attractive for 4D printing due to their predictable swelling behavior and compositional tunability. Common polymer platforms include chitosan-based, (Poly(ethylene glycol) Diacrylate) PEGMA-based, and PAA-based hydrogels, which enable programmable shape transformation when integrated into spatially patterned or multilayer printed structures [86,87,88,89].

For example, Wei Wang et al. [90] developed a pH-responsive interpenetrating Carboxymethyl Cellulose/Chitosan (CMC/CS) hydrogel that exhibited reversible swelling and deswelling across acidic, neutral, and basic environments. Such predictable pH-dependent volumetric changes are directly relevant to 4D-printed systems, where pH gradients can be exploited to program shape transformation or controlled actuation in chemically responsive environments.

Conductive polymers further extend the functionality of chemical-responsive 4D-printed hydrogels by coupling deformation with electrical or optical responses [91]. Ko et al. [92] developed pH-responsive (Polyaniline/Poly(ethylene glycol))PANI/PEG composite hydrogel arrays that exhibited reversible color changes under acidic and neutral conditions, enabling clear pH-dependent optical signaling. These characteristics are particularly relevant for 4D printing, where time-dependent changes in color or conductivity can be integrated into printed architectures for sensing, signaling, or adaptive device applications.

Despite the ease of preparation of the solutions to prepare pH-responsive hydrogels, pH-responsive hydrogels need a pH solution for their response, and they show a slow response time [93].

Ionic strength-responsive hydrogels undergo reversible swelling or contraction in response to specific ions or changes in overall ionic concentration, making them suitable for chemically programmable 4D-printed systems [94]. Luo and Sun demonstrated protein-based Ca^2+^-responsive hydrogels with ion-dependent mechanical modulation, highlighting the feasibility of ion-triggered property changes through specific molecular recognition [95]. Similarly, Di et al. reported a physically crosslinked PVA hydrogel with enhanced ionic conductivity and mechanical robustness achieved through salt-ion incorporation, enabling highly sensitive ionic responses [96].

Ionic strength-responsive hydrogels have also been explored for adaptive environmental applications. Heidy Cruz et al. [97] enhanced PAA hydrogels to achieve ion-dependent ammonium uptake, while Haidong Shi et al. [98] developed an anisotropic single-domain hydroxypropyl cellulose–PNIPAM hydrogel responsive to both temperature and ionic strength. Such anisotropic and multi-stimuli-responsive systems are particularly attractive for 4D printing, where directional swelling and ion-sensitive deformation can be programmed through printed architecture and material alignment.

Nevertheless, ionic strength–responsive hydrogels generally require ionic environments for activation and often exhibit moderate response rates, which can limit their applicability in rapidly actuating or large-scale 4D-printed systems [99,100].

### 3.4. Redox-Responsive Hydrogels

Redox-responsive hydrogels undergo reversible changes in structure or mechanical properties in response to oxidation–reduction reactions, enabled by functional groups such as disulfide bonds or redox-active moieties within the polymer network [101]. In the context of 4D printing, these materials allow time-dependent modulation of swelling, stiffness, or degradation when exposed to chemical or electrochemical stimuli, enabling programmable actuation and controlled release behaviors [102,103].

For example, Ismail Altinbasak et al. [104] developed PEG-based redox-responsive hydrogels incorporating disulfide-containing crosslinkers, where the redox state directly governed network density, porosity, and mechanical stability. By tuning crosslinker concentration, controllable swelling and degradation were achieved under reducing conditions, demonstrating how redox-triggered network reconfiguration can be leveraged in adaptive 4D-printed hydrogel structures.

**Figure 8:**
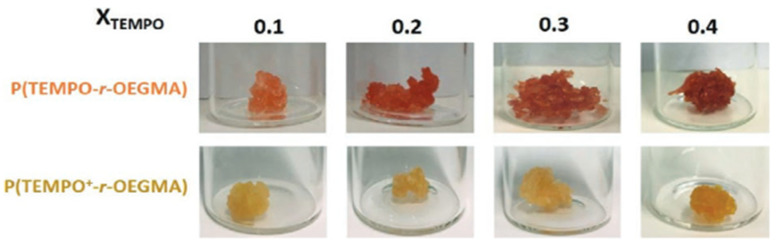
A collection of hydrogels composed of reduced P(TEMPO-r-OEGMA) and oxidized P(TEMPO^+^-r-OEGMA) was developed, featuring a crosslinking ratio (XCL) of 0.03 and a range of TEMPO compositions (XTEMPO) [105].

In another article, Mariam et al. [105] developed dual-responsive hydrogels combining temperature and redox sensitivity by incorporating nitroxide radicals and oligo(ethylene glycol) methacrylate into a single polymer network. These systems enable sequential or synergistic activation pathways, making them particularly attractive for 4D printing applications that require complex, time-dependent shape or property transformations. Figure 8 illustrates representative examples of such composite hydrogel systems.

Despite their potential, redox-responsive hydrogels face several challenges that limit their adoption in 4D printing, including limited mechanical and chemical stability, slow redox kinetics, and difficulty in precisely tuning redox potential [106,107]. Large volumetric changes during redox cycling can induce mechanical instability in printed structures, while the non-uniform distribution of redox-active species complicates fabrication and scalability. Additional constraints, such as biocompatibility concerns, environmental sensitivity, self-discharge effects, and high production costs, further restrict their use in practical 4D-printed devices [108,109].

### 3.5. pH-Responsive Hydrogels

pH-responsive hydrogels exhibit reversible swelling or deswelling behavior in response to environmental pH changes due to the ionization of functional groups such as carboxyl and amine moieties within the polymer network [110,111,112]. pH- and ion-responsive hydrogels rely on the density and spatial distribution of ionizable groups, which dictate osmotic pressure gradients, swelling ratios, and mechanical stability under stimulation [113,114]. In 4D-printed systems, this pH-dependent ionization enables chemically programmed, time-dependent deformation, where changes in chain charge density induce controlled volumetric expansion or contraction. Under acidic or basic conditions, protonation or deprotonation of these functional groups alters electrostatic interactions between polymer chains, resulting in predictable shape or property changes in printed architectures [43,115]. This mechanism provides a robust foundation for designing pH-triggered 4D-printed actuators, sensors, and adaptive structures operating in chemically heterogeneous environments.

**Figure 9:**
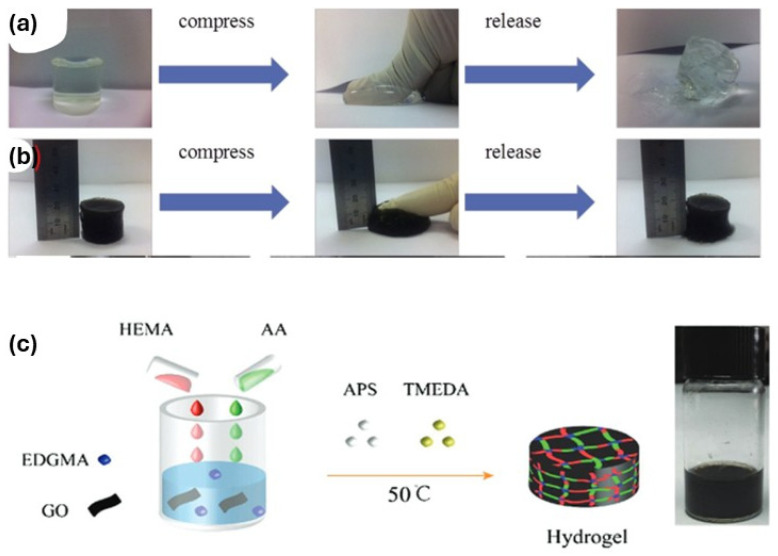
Macroscopic visualization of the deformation behavior was performed for (**a**) neat polyacrylic acid (PAA) and (**b**) its graphene composite hydrogel at 0.5 vol% [117]. (**c**) Synthesis of a pH-responsive conductive hydrogel composed of poly(acrylic acid-co-ethylene glycol dimethacrylate–hydroxyethyl methacrylate)/graphene oxide (poly(AA-co-EDGMA–HEMA)/GO) [117].

pH-responsive conductive hydrogels have attracted increasing attention for 4D-printed sensing and adaptive systems, as they couple chemical responsiveness with electrical functionality. Ma’s group reported a graphene-reinforced poly(acrylic acid) hydrogel that exhibited pH-dependent swelling behavior alongside enhanced electrical conductivity and mechanical robustness, demonstrating stable contraction under acidic conditions and expansion at higher pH values [116]. Figure 9a,b illustrates the deformation behavior of two hydrogels. In a related approach, a graphene oxide-based copolymeric hydrogel displayed reversible pH-triggered volume changes accompanied by significant conductivity modulation, transitioning from a contracted state in acidic environments to a swollen, more conductive state under basic conditions, as illustrated in Figure 9c [117]. While these systems highlight the potential of carbon-based fillers to enable simultaneous shape change and signal transduction, variations in mechanical characterization underscore the need for integrated material–structure design to ensure reliable performance in 4D-printed architectures.

Despite their versatility, pH-responsive hydrogels face several challenges that limit their application in 4D printing, including slow response times caused by diffusion-limited ion and water transport and insufficient mechanical strength that can lead to structural instability or delamination during repeated actuation [26]. Their effective pH window is often narrow and strongly influenced by temperature and ionic strength, complicating reliable shape programming. Conductive pH-responsive hydrogels frequently suffer from low conductivity and performance degradation under cyclic pH exposure, while complex fabrication routes and high material costs further hinder scalability and practical deployment [43].

### 3.6. Other Stimuli-Responsive Hydrogels

**Figure 10:**
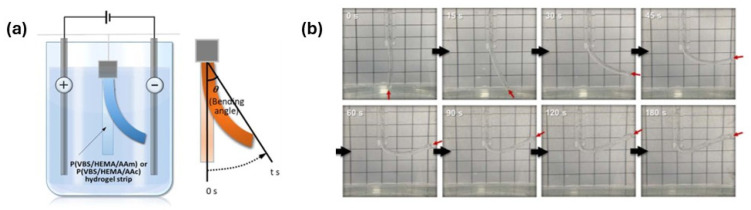
(**a**) A visual depiction of how a hydrogel bends when an electric field is applied. (**b**) The P(VBS/HEMA/AAm)-1/10/5 hydrogel-1.0, placed in a 0.025 M NaCl solution, shows clear bending motion under a 10 V electric field, showcasing its ability to respond to electrical stimulation [121].

Beyond moisture, thermal, and chemical stimuli, electrical fields can also induce swelling and deswelling in hydrogels, enabling fast and controllable actuation in 4D-printed systems. It is important to note that in light-, electric-, and magnetically responsive systems, the efficiency of stimulus transduction is modulated by the spatial distribution of photoactive, conductive, or magnetic components, as well as their interfacial interactions with the polymer matrix [118,119,120]. In their article, Yerin Shin et al. [121] demonstrated electro-responsive hydrogel actuators capable of rapid and reversible bending under low electric fields, where deformation behavior was governed by applied voltage, electrolyte ionic strength, crosslinking density, and polymer composition. Figure 10a,b illustrates how a hydrogel bends when an electric field is applied. Such electroactive hydrogels are particularly attractive for 4D-printed soft robotic and adaptive devices due to their high response speed and precise controllability. However, their practical implementation is constrained by the requirement for external electrodes and electrolyte environments, which complicates device integration and scalability [122].

Some hydrogels respond to externally applied magnetic fields. Such hydrogels fall under the category of magnetic responsive hydrogels. In an article related to such stimuli, Daria Podstawczy et al. [123] developed 3D-printed stimuli-responsive actuators made from magnetic nanoparticle-embedded alginate–methylcellulose hydrogels. A thixotropic ink for magnetic 3D printing was formulated using alginate and methylcellulose, incorporating PAA-stabilized magnetic nanoparticles using the DIW 3D printing technique. It was observed that while methylcellulose enhanced the ink’s rheological properties, it negatively affected the mechanical stability of the hydrogel. One advantage of using magnetic response hydrogels is to control them remotely; however, this sort of response is only achieved with magnetic particles [124].

Combining multiple distinct stimuli enhances the versatility and applicability of hydrogels. In such an attempt, Saeeun Jang et al. [56] developed a 4D-printed untethered milli-gripper made from a biodegradable and biocompatible electro- and magneto-active hydrogel. In this study, an electromagnetic field-responsive untethered milli-gripper was developed using a bio-3D printer. The device incorporated a biocompatible and biodegradable chitosan hydrogel, enhanced with citric acid-coated SPIONs. When an electric field is applied in an electrolyte, the untethered milli-gripper undergoes deswelling toward the positive electrode (anode), causing it to bend in that direction and creating a gripping motion. The presence of citric acid-coated SPIONs allows for precise 3D positioning of the milli-gripper using a neodymium permanent magnet. In another paper related to multiple responsive hydrogels, Xinmeng Zhou et al. [125] developed triple-responsive (Glucose, Temperature, and pH) nanocomposite hydrogel microneedles for controllable drug delivery through UV-cured photopolymerization. The ink formulation consists of four functionally diverse monomers: 2-(dimethylamino)ethyl methacrylate, N-isopropyl acrylamide, acrylic acid, and acrylamide. These monomers are crosslinked using aluminum hydroxide nanoparticles, which act as both reinforcing agents and crosslinking centers. This results in a nanocomposite hydrogel with exceptional mechanical strength, which is essential for the 3D printing of hydrogel microneedle patches.

Light-responsive hydrogels are smart materials that react to external light stimuli. Upon exposure to light, they shrink by expelling water, allowing for non-invasive, cost-effective, and remotely controlled actuation. Emilia Zari et al. [57] present a light-driven, 3D (Extrusion)-printed elastomer/hydrogel composite actuator. The soft photo-actuator integrates TangoPlus, a flexible 3D printing material, with a PNIPAM hydrogel copolymerized with the photochromic molecule spiropyran. The composite actuator’s passive layer is composed of TangoPlus, a flexible 3D printing material. This elastomeric layer enhances the mechanical integrity of the hydrogel-based actuator. The incorporation of the spiropyran element into the PNIPAM gel makes it photo responsive. In another article, Qijun Wu et al. [126] described the creation of a light-responsive actuator by integrating tissue paper with a hydrogel composite made from PNIPAM. This was accomplished through an uncomplicated in situ polymerization method that involved inkjet-printed tissue paper. The prepolymer solution was formulated by dissolving N-Isopropylacrylamide (NIPAM) (the monomer), N,N’-Methylenebis(acrylamide) as the crosslinker, and 2,2′-Azobis(2-methylpropionamidine) dihydrochloride as the PI in deionized water. The composite actuator benefited from the inherent strength of natural tissue paper, coupled with strong interactions at the interface of the bilayer structure, which collectively enhanced its mechanical properties and resulted in a tensile strength of 1.2 MPa. Light-driven hydrogels can be remotely controlled, but it is difficult to control light intensity penetration depth [127].

**Table 1:** Commonly reported stimuli-responsive hydrogel matrices: types, fabrication techniques, and applications.

Stimulus	Materials Responsible for Shape-Morphing	Material (Hydrogel: Composition of the Base Material)	Fabrication Technique	Swelling	Response Speed	Reversibility	Proposed Application(s)	Reference
Temperature	poly(NIPAM-co-DMAPMA)/clay	Bilayer NIPAM + DMAPMA + crosslinking agent (MBA) + light initiator TPO + rheological modifier (Laponite XLG)	DIW	Moderate	Fast	High	Bionic	[128]
Temperature	PVA/(PVA-MA)-g-PNIPAM	PVA + NIPAM + Photoabsorber (tartrazineas) + light initiator TPO	DLP	Moderate	Fast	High	Actuators	[72]
Temperature	PAA	Acrylic Acid + PI (TPO) + crosslinker (dexadecyl acrylate)	SLA	High	Slow	High	Biomedical	[129]
Temperature	PNIPAM PNIPAM + PiPrOx	2-isopropyl-2-oxazoline + 2-Methyl-2-oxazoline + NIPAM + PI (TPO) + photoabsorber (Orange G)	SLA	Moderate	Fast	High	Biomedical	[130]
Temperature + pH	NIPAAm + MA-BSA	poly(ethyleneoxide)-b-poly(propylene oxide)-b-poly(ethylene oxide) + Photocurer (Lithium phenyl-2,4,6-trimethylbenzoylphosphinate)	DIW	High	Moderate	Moderate	Biomaterials	[25]
Temperature	PNIPAM + Alginate	NIPAAm + crosslinker (PEGDA) + PI + rheological modifier (Laponite XLG)	-	Moderate	Moderate	High	soft robotics	[131]
Temperature	PNIPAM	NIPAM + PI a-ketoglutaric acid + cross-linker(MBA) + Rheology modifier Carbomer 940	Extrusion + UV curing	Moderate	Slow–moderate	High	Drug release	[132]
Temperature + Light	PNIPAM + Prussian Blue Nanoparticles	(NIPAM + PEG dimethacrylate) + crosslinker (PEGDA700) + PI (Darocur 1173)	SLA	Moderate	Fast	High	Actuators	[133]
Temperature	(PNIPA/PAA)	rheological modifier (Laponite) + NIPAM + Crosslinker N,N′-methylenebisacrylamide (Bis) + PI (LAP)	Extrusion	High	Moderate	High	Actuators	[134]
Temperature	PNIPAM/Alginate/CNF	NIPAM + PI (Irgacure) + crosslinker (MBA) + Crosslinker PEGDA + Reinforced (TCNF)	DIW	Moderate	Moderate	High	Drug Release	[135]
Temperature	PNIPAM	NIPAM + crosslinker (MBA) + rheology modifier (Carbomer)	DIW	Moderate	Moderate	High	controlled drug release	[136]
Humidity	poly(MAA-co- OEGMA)	oligo(ethylene glycol) methacrylate (OEGMA) + ethacrylic acid (MAA) + 4-Nitrophenyl benzoate (Catalyst)	DIW	High	Slow	Moderate	soft robots	[137]
Humidity	PLA + PHBV	Bilayer Polyurethane + polyketone + PLA	FFF	Low–moderate	Slow	Moderate	Smart structures	[138]
Temperature + Hydration	Poly(N-vinyl caprolactam) (PNVCL)	Bilayer N,N-Dimethylacrylamid + NVCL + Crosslinker (PEGFMA) + PI (Irgacure)	SLA	Moderate	Moderate	High	actuator	[139]
Ca^2+^/chitosan	Sodium Alginate	Sodium alginate + 2-(Dimethylamino) ethyl methacrylate + methacrylic anhydride + PI 2-hydroxy-2-methylpropiophenone	DIW	High	Slow	Moderate	-	[140]
Humidity	Chitosan + Acetic Acid	Chitosan Powder + Crosslinker (Critric Acid) + Rheological Modifier (trimethyl silane spray)	DIW	High	Slow	Moderate	Actuator	[141]
Water	Non-isocyanate poly(hydroxyurethane)	poly(ethyleneglycol) + Chain Extender poly(ethylene oxide) diamine + cross-linker tris(2-aminoethylene)amine (TAEA)	DIW	High	Slow	High	Biomedical	[142]
Temperature + Water	polyurethane (PU)-polyvinyl chloride (SMPVC) bilayer	Bilayer Water swelling (PU) + Heat shrinkage shape (SMPVC)	DIW	Moderate	Moderate	High	-	[143]
pH of acidic or basic Environment	PAAm/PAAc	Bilayer Acrylic acid + Crosslinker (MBA) + UV initiator (ammonium persulfate) + coupling agent ((trimethoxysilyl) propylmethacrylate) + cyanoacrylate adhesive to bond two layers	-	High	Slow	Moderate	Lipophilic Drug Delivery	[144]
Magnet	Magnetic hydrogel structures from natural polymers	alginate (ALG) + methylcellulose (MC) + polyacrylic acid (PAA) + magnetite nanoparticles of Fe_3_O_4_ (MNPs) + 0.5 M CaCl_2_ solution for 24 h for Ca^2+^ crosslinking	DIW	Low	Fast	High	Actuators	[38]
Temperature	PNIPAM/short carbon fibers (SCFs)	NIPAM + PI (2959) SCFs + Rheology modifier (clay nanosheets) + (PEGDA, PEGDA) + catalyst (N,N,N’,N’-Tetramethylethylenediamine) (TEMED) + Glucose + glucose oxidase	DIW	Moderate	Fast	High	Self-Sensing Actuators	[145]
pH	poly(4-vinylpyridine) (P4VP) + Acrylonitrile butadiene styrene (ABS)	Pure ABS + P4VP + 10 mM phosphateecitrate (for pH sensing) + Ammonium acetate	FFF	Low–moderate	Slow	Moderate	Sensing claw	[146]
Temperature	PNIPAM/gold nanorod	NIPAm monomer + PI (Irgacure 819) + ethylene glycol/acetone solution + macrocrosslinkers (bi-, tri-, and tetra-allyl-functional PNIPAm)	Multiphoton lithography	Moderate	Fast	High	bioinspired soft materials	[147]
Dehydration	Ceramic elastomer slurry/acrylic acid-PEGDA (AP) precursor with low viscosity	Crosslinker (PEGDA) + Ceramic elastomer: benzyl acrylate (BA), PEGDA and zirconia (ZrO_2_) nanopowders	DLP	Low	Slow	Moderate	-	[148]
Light	carbon nanotube-doped NIPAM composite (CNNC)	NIPAM + (Single wall CNTs) SWNTs with polyaniline sulfonic acid groups + cross-linking agent (MBA) + PI (Triethanolamine + LAP)	femtosecond laser direct writing	Moderate	Fast	High	microbots	[149]
Magnet	Light-curable magnetic hydrogel elastomer (PLMHE) with magnetic controllability	Monomer PEG (4 0 0) DA + catalyst (OMNIRAD TPO) + Thickening agent (Bentonite clay) + neodymium iron boron magnetic powder + Mechanical properties enhancer (Polyethylene glycol) + Light initiator (TPO)	Extrusion	Low	Fast	High	Soft Actuators	[150]
Water	Acrylic acid (AAC) network and Fe^3+^ ions	Acrylic acid (AAC) + Physical crosslinkers (Fe^3+^) + cross-linker (MBA) + PI (LAP)	DLP	High	Slow	Moderate	Stretchable electronics	[151]
Water + Light	Liquid metal nanodroplets armored by carbon dots (LMD@CDs) + poly acrylamide (AAm)	Monomer (AAm) + PI (TPO) + cross-linker (PEGDA) + Carbon-dot-armored liquid metal nanodroplets (LMD@CDs)	DLP	Moderate	Fast	High	-	[152]
Dehydration + Rehydration	F-127-based hydrogel	Photo absorber (Tartrazine) + F-127 diacrylate + PI (LAP+ Irgacure 2959) + LiCl solution	DLP	High	Moderate	Moderate	Strain Sensor	[153]
Temperature + Light	(NIPAm) and polyethylene glycol thiol-coated gold nanorod (P-AuNR) hydrogel	NIPAm + P-AuNR + AAm + chemical crosslinking agent (N,N-Methylenebisacrylamide) + UV PI (irgacure 295)	Photopolymerization	Moderate	Fast	High	-	[154]

Swelling is classified qualitatively as: low (<100% volume increase), moderate (≈100–400%), and high (>400–500%) based on equilibrium volumetric expansion or swelling ratios reported.

## 4. Applications

**Figure 11:**
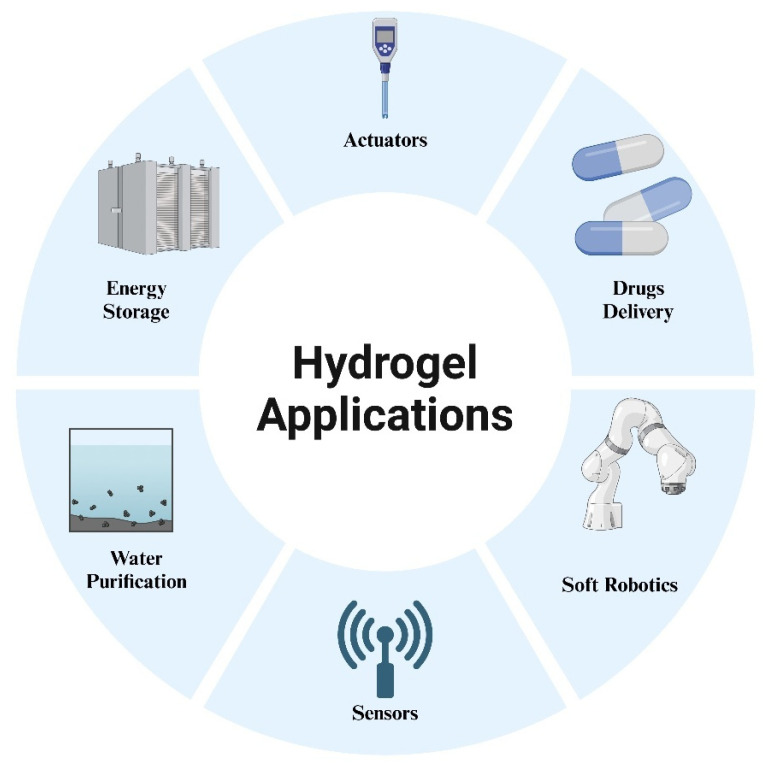
Key applications of hydrogels.

Hydrogels have been widely investigated for decades; however, their integration with 4D printing has shifted their role from static materials to adaptive systems capable of time-dependent shape and property transformation [155]. By combining stimuli-responsive material design with additive manufacturing, hydrogels can be programmed to undergo controlled deformation, reconfiguration, or functional changes after printing. This section focuses on representative application areas where 4D-printed hydrogels provide clear advantages over conventional hydrogel systems, particularly in actuation, water treatment, and sensing [156]. Figure 11 illustrates some key applications of hydrogels.

### 4.1. Hydrogel-Based Actuation and Soft Robotics

Soft actuators and robots represent one of the most mature application areas of 4D-printed hydrogels, where stimulus-responsive deformation enables compliant and adaptive motion in delicate environments [157]. In hydrogel-based 4D-printed actuators, deformation is typically activated by external stimuli such as temperature, humidity, light, or magnetic fields, which trigger programmed swelling, contraction, or stiffness changes in the printed architecture [158,159,160,161]. Hydrogels have attracted considerable interest in soft robotics due to their softness, flexibility, biocompatibility, and stimuli responsiveness. Their compliant, continuously deformable structures enable safe human–robot interaction and allow complex, adaptable motion that is difficult to achieve with rigid robotic systems, making them well suited for actuators and sensors in soft robotic applications [162]. In soft robotic systems, printed hydrogels are commonly actuated using thermal, optical, magnetic, or electrical stimuli to enable controlled locomotion, gripping, or reconfiguration [121,163,164].

**Figure 12:**
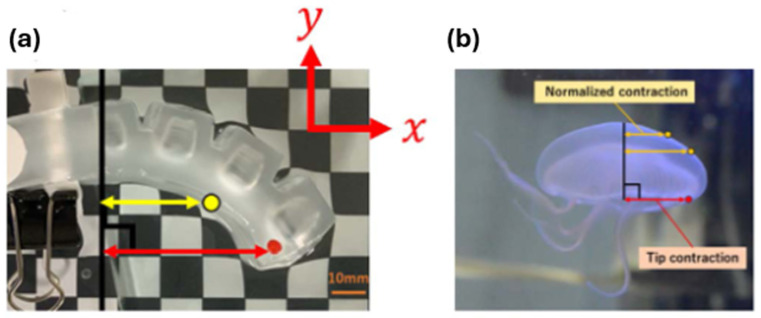
Images showing the measured points of (**a**) a 3D-printed actuator and (**b**) a moon jellyfish [165].

In a paper related to actuators, Yuki Takishima et al. [165] reported the creation and analysis of a 3D-printed hydrogel soft actuator, designed to resemble a jellyfish. These actuators were composed of three components: (1) Connector, serving as the junction between the main body of the actuator and the air pressure inlet tube, (2) Box, functioning as the balloon-like inflation section, and (3) Base, which is attached to the Box. Figure 12a,b presents images showing the measured points of actuators. A bilayer hydrogel actuator with controllable temperature and near-infrared laser responses was fabricated by Qian Zhao et al. [166]. Enhanced bonding strength in hydrogel actuators was achieved through polymerization at the interface boundary and anisotropic microstructures. The integration of molding and 3D printing facilitated the creation of bilayer hydrogel actuators characterized by enhanced mechanical strength and responsive deformation to temperature changes and infrared laser stimuli.

**Figure 13:**
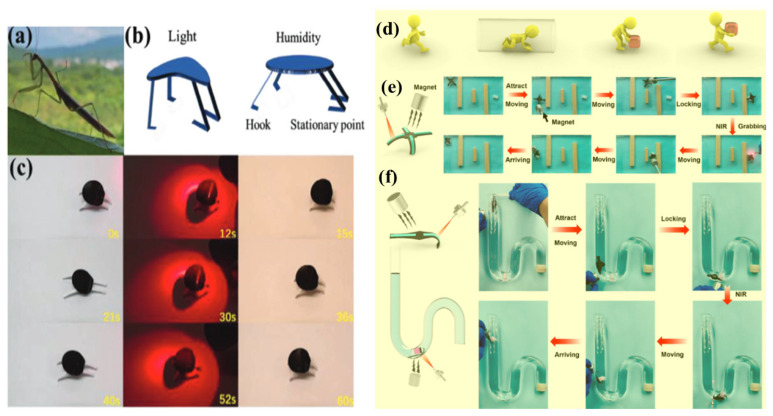
Smart multifunctional uses of WMR actuators mimicking nature with biomimetic walkers, intelligent curtains, adaptive grippers, and rolling mechanisms. (**a**) Snapshot of a praying mantis in the wild. (**b**) Diagram showing biomimetic walkers bending under light and flattening under humidity. (**c**) Photos capturing the walker’s forward motion powered by alternating light and humidity triggers [167,168]. (**d**) Diagram showing the soft robot’s ability to move, overcome obstacles, grab, and transport target objects. (**e**) Design of a four-claw soft actuator that navigates narrow gaps with magnetic guidance, grips objects under near-infrared (NIR) light, and carries them away. (**f**) Structure of a two-claw soft actuator, with visuals demonstrating its capability to enter a thin tube, retrieve objects, and bring them to the surface [168].

Inspired by the locomotion of praying mantises, a biomimetic walker was developed using a 3D-walking magnetic robot actuator integrated with simple printed substrates, demonstrating how bioinspired motion can be achieved through efficient design and accessible materials [167]. The 3D-walking magnetic robot actuator was constructed by integrating a magnetic elastomer layer with a hygroscopic substrate and bioinspired leg structures. Under alternating light and humidity stimuli, the system exhibited continuous forward crawling driven by asymmetric friction between the front and rear limbs, demonstrating how multi-stimuli-responsive materials can be programmed to generate autonomous, bioinspired locomotion. Figure 13a–c represents the smart multifunctional uses of WMR actuators. Han et al. [168] developed a double-layer hydrogel actuator by combining inert gelatin infused with a shape-memory hydrogel [tannic acid gelatin (TAG)] and Fe_3_O_4_ particles, resulting in a material capable of dual responses to temperature and light. Building on this, they designed a remote-controlled soft robot that leverages light-induced shape transformation and magnetic field-guided motion for precise control and movement. The robot is capable of navigating through winding spaces, grasping target objects, and retrieving them, as illustrated in Figure 13d–f. Guided by external magnetic fields, both four-claw and double-claw robots demonstrate controlled navigation in confined environments, while near-infrared light triggers grasping and release, enabling programmable capture and transport of objects through combined magnetic and optical actuation.

A key challenge for soft robotics is achieving reliable actuation while minimizing environmental risks associated with non-biodegradable or hazardous materials. To address this challenge, Sun et al. [169] demonstrated the use of free-form reversible embedding in suspended hydrogels through 3D DIW to create small-scale, biologically derived hydraulic actuators capable of generating millinewton-level forces with complex actuation geometries. This innovative printing technique facilitates the creation of intricate robotic structures in a single step, removing the necessity for multi-stage casting or the assembly of separate actuators post-fabrication. Similarly, in another article, Ellen H. Rumley et al. [170] created sustainable soft robots using biodegradable electrohydraulic actuators. These actuators were developed using a range of materials, including various biodegradable polymer films, an ester-based liquid dielectric, and a gelatin hydrogel infused with NaCl. Collectively, these studies highlight that biodegradable, and bio-derived hydrogels can match the performance of conventional materials while offering safer and more sustainable pathways for 4D-printed soft robotic systems.

Although hydrogels are attractive actuator and soft robotics materials due to their biocompatibility and multi-stimuli responsiveness, their application in 4D-printed systems is limited by diffusion-controlled response speed, low actuation force, restricted deformation range, and sensitivity to environmental conditions. Current research, therefore, focuses on material reinforcement, network engineering, and architectural design to improve actuation efficiency, durability, and functional versatility in 4D-printed hydrogel actuators [171]. In addition to conventional hydrogel systems, emerging materials are increasingly being explored to overcome limitations in force output, response speed, and durability. These include double-network and interpenetrating hydrogels, nanocomposite hydrogels reinforced with nanocellulose, graphene, or magnetic nanoparticles, and supramolecular or dynamic covalent hydrogels that enable self-healing and fatigue resistance [159,172,173]. Hybrid systems that integrate hydrogels with elastomers, liquid metals, or conductive polymers are also gaining attention, as they combine large deformation with enhanced mechanical robustness and multifunctionality, making them promising candidates for next-generation 4D-printed actuators [160,174].

### 4.2. Energy Storage

Hydrogels have gained attention in energy conversion and storage due to their porous structure, tunable physicochemical properties, and high ionic conductivity [175,176]. Their water-rich, interconnected networks provide efficient ion transport, making hydrogel electrolytes suitable for flexible batteries and supercapacitors [177].

In energy storage applications, 4D-printed and deformable hydrogel systems are primarily activated by electrical and electrochemical stimuli, where changes in ion transport, conductivity, or electrode–electrolyte interfaces govern device performance. In this context, graphene-based hydrogel fibers and networks have been explored as flexible electrodes or conductive scaffolds due to their combined electrical conductivity, mechanical compliance, and processability. Graphene-based hydrogels are frequently engineered into 1D fiber or strand-like geometries to leverage their high aspect ratios and mechanical flexibility [178]. This structural choice specifically facilitates their use in wearable and flexible energy storage devices (e.g., supercapacitors) [179,180]. In an article, Chaojun Wang et al. [181] developed drying graphene hydrogel fibers for capacitive energy storage. Different dried graphene fibers exhibited a wide range of specific volumetric capacitance, from 5 to 120 F cm^−3^, and diverse rate capabilities in capacitive energy storage.

**Figure 14:**
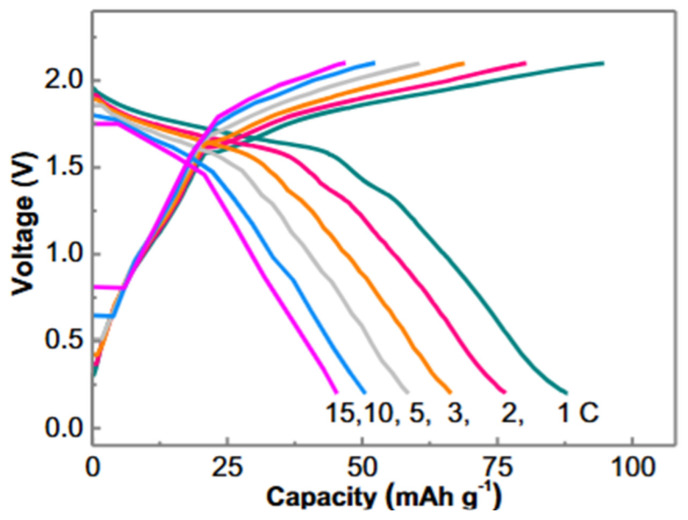
Galvanostatic charge–discharge profiles of a Zn‖MnHCF full cell using a lean-water hydrogel electrolyte, demonstrating stable voltage plateaus and sloping regions associated with a combined battery-type and pseudocapacitive charge-storage mechanism [183].

Similarly, in another article, Minfeng Chen et al. [182] developed a versatile hydrogel electrolyte tailored for environmentally adaptive, dendrite-free aqueous Zn‖MnO_2_ batteries. This innovative hydrogel electrolyte demonstrated impressive ionic conductivity, enhanced mechanical properties including high tensile strength and elasticity, an exceptionally low freezing point, effective self-healing capabilities, robust adhesion, and outstanding heat resistance. In another article related to hydrogel electrolytes, Yanbo Wang et al. [183] created solid polymer and hydrogel electrolytes for use in zinc-ion batteries. The hydrogel electrolyte demonstrated high ionic conductivity along with remarkable mechanical properties, including impressive tensile strength and elasticity. It also featured an exceptionally low freezing point, effective self-healing capabilities, strong adhesion, and excellent heat resistance. Figure 14 illustrates the electrochemical performance of a Zn‖MnHCF cell. Flexible supercapacitors are emerging as attractive energy storage solutions because of their portability and extended cycle life. Nonetheless, their practical use is limited by inadequate interfacial adhesion between the layers of the supercapacitors. To address this problem, Dingkun Wang et al. [184] introduced lignin-infused hydrogel matrices that exhibit improved adhesion and toughness for application in all-hydrogel supercapacitors. This research details the creation of a hydrogel matrix formed by incorporating Ag-lignin nanoparticles into a polyacrylamide network. The distinctive interwoven microfibril architecture and the non-covalent interactions within the matrix lead to a notable increase in both adhesion and mechanical strength. Jiahui Zhao et al. [185] developed a double-cross-linked hydrogel composed of PANI and PVA, known as a PH-A hydrogel. The dual-crosslinking approach aimed to improve the hydrogel’s mechanical properties and electrochemical performance, making it a promising candidate for energy storage applications. In another article where hydrogel was used as an electrolyte, Jian-hao Lin et al. [186] made a self-healable and redox-active hydrogel electrolyte for supercapacitor applications by incorporating ferric ions. The redox reaction of Fe^3+^ occurred on polyaniline/CNT electroactive material, resulting in a high capacitance and remarkable energy density, along with excellent cycling stability. Furthermore, the hydrogel could regain its electrochemical performance after being cut and healed, significantly improving the reliability and safety of the energy storage device.

Bio-waste-derived hydrogels have recently emerged as sustainable and high-performance materials for supercapacitor applications, offering advantages such as low cost, biodegradability, and tunable physicochemical properties. Lignin, a redox-active biopolymer, has been incorporated into conductive hydrogel networks to enhance charge storage capabilities through its quinone/hydroquinone redox couples, as demonstrated by [184] in lignin-Ag nanoparticle-reinforced hydrogels for all-hydrogel supercapacitors with improved adhesion and cycling stability. Demonstrating the expanding multifunctional potential of these materials, Muddasar et al. [187] recently developed the first fully lignin-derived ionic thermoelectric supercapacitor. This pioneering device integrates low-grade thermal energy harvesting and electrochemical storage into one platform, using a crosslinked lignin hydrogel electrolyte (Seebeck coefficient of 9.4 mV/K, ionic conductivity of 93.63 mS/cm) paired with carbonized lignin electrodes. A notable study by Zhang et al. [188] engineered a cellulose nanofiber (CNF)-based double-network hydrogel electrolyte, achieving an ultra-high ionic conductivity of 103 mS cm^−1^ and enabling a wide operating voltage of 1.8 V for flexible supercapacitors by introducing redox-active K_3_[Fe(CN)_6_]. In a parallel development, Li et al. [189] successfully fabricated a Carbon (C)/Polyacrylamide (PAM) Interpenetrating Polymer Network (IPN) hydrogel electrolyte, which achieved an exceptional ionic conductivity of 131.4 mS cm^−1^ and enabled a flexible supercapacitor with a high areal capacitance of 989 mF cm^−2^. This work underscores the potential of cellulose-based double-network structures in providing both robust mechanical support and efficient ion transport channels. Chitosan–graphene oxide composite hydrogels prepared via green synthesis routes have also shown remarkable capacitive performance [190]. Notably, when the hydrogel electrode was paired with a lignin hydrogel electrolyte to assemble an all-solid-state flexible supercapacitor, it achieved an ultrahigh energy density of 31 Wh/kg, significantly surpassing many similar devices, and demonstrated potential for application as a signal sensor. These advances underscore the potential of bio-waste hydrogels in developing environmentally benign, high-performance, and increasingly multifunctional energy storage systems.

However, there are some limitations; for instance, in supercapacitors with ultrahigh flexibility, the main challenge lies in the poor interfacial adhesion of hydrogel-based electrode/electrolyte interfaces, leading to detachment during deformation and hindering electrochemical performance. The water-decomposition issue in current supercapacitors limits their electrochemical performance, including output voltage and energy density [191].

### 4.3. Water Purification

**Figure 15:**
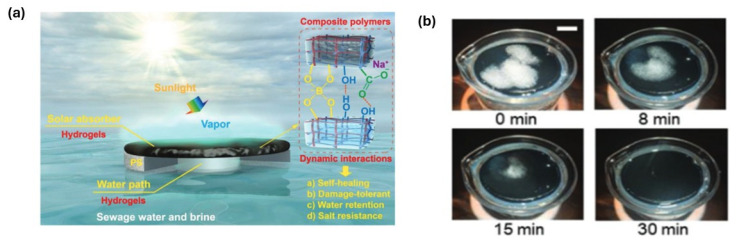
(**a**) Schematic representation and working mechanism of the interfacial steam generator proposed [195]. (**b**) Evaporation and anti-salt-fouling performance of hydrogel under sun illumination, with initial salt crystallization presence [196].

Hydrogels are well suited for water purification due to their hydrophilicity, swellability, and porous network structure, which can be exploited for interfacial solar evaporation and desalination [192,193]. Youhong Guo et al. [194] created highly elastic interconnected porous hydrogels through a self-assembled templating method aimed at solar water purification. This approach proved to be a simple and effective technique for fabricating large-scale elastic hydrogel evaporators that exhibit outstanding desalination performance. In another similar article, Fangbin Li et al. [195] made self-repairing and damage-tolerant hydrogels designed for efficient solar-powered water purification and desalination. A durable, monolithic, and self-floating interfacial steam generator was successfully created through the simple integration of self-healing polymeric hydrogels, demonstrating high-performance solar-driven water evaporation and desalination. Figure 15a presents the working mechanism of the interfacial steam generator proposed in this study. Youhong Guo et al. [196] developed biomass-derived hybrid hydrogel evaporators tailored for affordable solar water purification. The hybrid hydrogel evaporators exhibited efficient water transport, effective water activation, and anti-salt-fouling properties, leading to a high evaporation rate. Figure 15b represents the evaporation and anti-salt-fouling performance of hydrogel.

Advanced technologies and materials are essential for removing pollutants from contaminated water. The adsorption process offers numerous advantages, including high adaptability and removal efficiency for water sources of varying quality [197]. Zheng et al. [198] synthesized a hydrogel incorporating silver nanoparticles, porphyrin, and reduced GO, designed specifically as a dye adsorbent for wastewater treatment. Gao-Jie Jiao et al. [199] successfully removed heavy metal ions from wastewater for reuse in chemiluminescence through the successive application of lignin-based composite hydrogels. In this work, a sulfomethylated lignin-grafted-polyacrylic acid hydrogel was fabricated using a simple and environmentally friendly synthetic strategy. In another article, Van Thuan Le et al. [200] developed eco-friendly cellulose-based hydrogels intended for water treatment and purification. Despite their promise, challenges remain, including relatively low mechanical strength and durability. In another study, Md. Samrat Hossain et al. [201] developed a hydrogel-based superabsorbent for the efficient removal of heavy metals in industrial wastewater treatment and environmental conservation. Yan Liu et al. [202] created granular composite hydrogels designed for the recovery of ammonia nitrogen from wastewater, with the goal of enhancing crop growth. Aurel Diacon et al. [203] created dual-responsive hydrogels designed for detecting and removing mercury ions from wastewater.

While hydrogels are an effective method for wastewater purification, their regeneration capacity is often restricted. After several treatment cycles, these adsorbents can lose their active sites, resulting in waste and potential secondary pollution. Hence, it is essential to tackle this challenge sustainably. Innovative research is required to establish a circular approach that enhances the value of spent adsorbents through reusability. One possible solution is to repurpose spent adsorbents for other applications, such as catalysis, antimicrobial uses, or energy applications, to achieve zero or minimal waste generation [204].

### 4.4. Sensors

Advances in materials chemistry and processing have enabled the functionalization of hydrogels for adaptive and multi-functional sensing [205]. For sensing applications, external stimuli such as strain, pH, humidity, or temperature are applied to printed hydrogels, inducing measurable changes in electrical, optical, or mechanical properties [92,117].

Flexible wearable devices are attracting considerable interest due to their benefits of wearability, compact design, and adaptability [206]. To gain advantages from these properties, Dequan Wei et al. [207] created multifunctional bi-network ionic conductive hydrogels that utilize on-demand graft modification, enabling real-time monitoring of human movements through wearable sensors.

**Figure 16:**
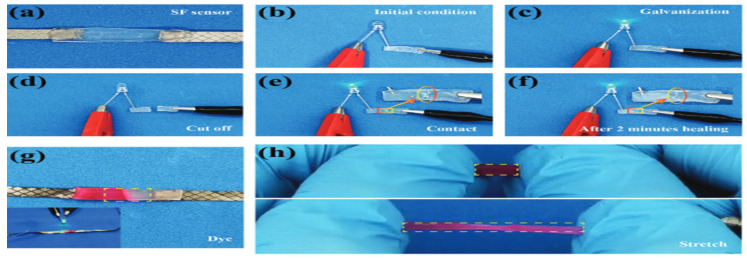
Demonstration of self-healing capability after being severed and rejoined. (**a**) Initial state of the hydrogel. (**b**) Integrated into an active circuit. (**c**) Illuminates an LED when powered by 3.0 V. (**d**) Hydrogel is cut into two separate pieces. (**e**) The LED reignites at the same voltage after reconnection. (**f**) After two minutes of healing, the junction crack becomes less visible. (**g**) One segment is dyed, with the insect confirming the SFH remains conductive. (**h**) After 12 h of healing, the dye diffuses through the junction, resulting in a uniform color, and the restored SFH retains its stretchability [208].

In another article, Li Zhao et al. [208] created ultra-stretchable, adhesive, and self-repairing hydrogels infused with silk fibroin for use in wearable sensors. A straightforward one-pot thermal polymerization method is introduced to fabricate silk fibroin-doped hydrogels (SFH). These hydrogels are both chemically and physically cross-linked using acrylamide, acrylic acid, and silk fibroin. In conclusion, the SFH has extensive applications in various fields, including electronic skin, soft robotics, wearable electronic devices, vocal cord disease monitoring, and human–machine interaction. Figure 16 demonstrates the self-healing capability after being severed and rejoined.

Conventional hydrogel strain sensors are often limited by low stretchability and high hysteresis, restricting their effectiveness in wearable and robotic applications. To overcome these limitations, Zequn Shen et al. [209] developed highly stretchable, ultra-low-hysteresis conductive hydrogel sensors using a microphase semi-separated network that integrates PEDOT nanofibers with a PVA matrix, significantly improving mechanical resilience and signal stability. Beyond isotropic sensing, anisotropic conductive hydrogels have been introduced to better mimic the directional properties of biological tissues. Such systems, achieved through controlled alignment strategies, exhibit direction-dependent mechanical and sensing responses, offering enhanced functionality for 4D-printed sensors and soft robotic components where spatially programmed and adaptive sensing is required.

The development of underwater strain sensors remains a significant challenge due to the swelling of hydrogels in aquatic environments. Jiayuan Ren et al. [210] developed an anti-swellable hydrogel strain sensor designed for underwater motion detection. The fabrication involved creating an anti-swellable hydrogel composed of PVA, a copolymer of [2-(methacryloyloxy) ethyl] dimethyl-(3-sulfopropyl) ammonium hydroxide, and 2-hydroxyethyl methacrylate. High sensitivity was achieved, enabling the detection of multidirectional motions such as raising the head, swinging the arm, and bending the elbow, knee, and finger. Li Chen et al. [211] developed a supramolecular polyionic liquid hydrogel characterized by its anti-swelling properties, specifically tailored for underwater sensing applications. The hydrogel incorporated an ionic liquid with an extended alkyl chain, which created a hydrophobic region within the structure. This design significantly reduced the swelling ratio and limited ion diffusion, enhancing the hydrogel’s performance in aquatic environments. Xiaoqing Ming et al. [212] crafted anti-swelling conductive polyampholyte hydrogels using ionic complexation, specifically tailored for applications in underwater motion sensing and dynamic information storage. This hydrogel, derived from a copolymer of acrylic acid and 1-butyl-3-vinylimidazolium bromide, exhibits remarkable capabilities in accurately and consistently sensing electromechanical responses across a broad range of strains. As a result, it serves as an excellent candidate for strain sensors designed to monitor human movements effectively in both air and aquatic environments.

### 4.5. Drug Delivery

Owing to their high water content, biocompatibility, and tunable properties, hydrogels have become a prominent platform for drug delivery. Their three-dimensional, cross-linked networks enable the encapsulation of diverse therapeutic agents—from small molecules to proteins and nucleic acids—facilitating controlled and sustained release. Their ability to respond to environmental stimuli, such as pH, temperature, light, or enzymatic activity, makes them particularly attractive for targeted and stimuli-responsive drug delivery systems [213]. In addition to controlled drug release, the degradation behavior and osteogenic potential of hydrogel-based materials are critical constraints in regenerative applications, particularly in bone tissue engineering, where scaffold resorption must be synchronized with new tissue formation. Comprehensive analyses of chitosan- and hydrogel-based 3D-printed constructs have shown that degradation kinetics, crosslinking density, and ionic composition jointly regulate mechanical stability, mineral deposition, and osteogenic differentiation, rather than acting as independent design variables [214]. Recent studies [215,216] have demonstrated that hydrogel degradation kinetics, crosslinking density, and ionic composition strongly influence bone regeneration, mineralization, and tissue integration, highlighting the importance of coupling material design with biological performance in degradable hydrogel systems.

A key advantage of hydrogels is their stimuli-responsive drug release capability, which is highly suitable for personalized medicine. Temperature-sensitive variants, like those incorporating PNIPAm, utilize a phase transition at their lower critical solution temperature (LCST) to trigger release at physiological temperatures [217]. This property is highly beneficial for cancer therapy, as localized hyperthermia can be used to trigger the release of chemotherapeutics directly at the tumor site, thereby improving efficacy and reducing systemic side effects. Likewise, pH-sensitive hydrogels—frequently made from PAA or chitosan—are engineered to release drugs in acidic microenvironments like those in tumors or the stomach, making them well-suited for targeted cancer and gastrointestinal treatments [218]. Light-responsive hydrogels, which incorporate photochromic molecules or nanoparticles, offer another layer of control, allowing for precise, on-demand drug release when exposed to specific wavelengths of light [219]. Furthermore, enzyme-responsive hydrogels can be engineered to degrade and release their payload in the presence of specific enzymes overexpressed in pathological conditions, such as matrix metalloproteinases (MMPs) in tumor microenvironments [220]. These stimuli-responsive properties enable precise spatiotemporal control over drug release, which enhances therapeutic efficacy while minimizing adverse effects. The integration of multiple responsive mechanisms within a single hydrogel platform further augments this precision and versatility, paving the way for advanced, multifunctional therapeutics.

Despite their numerous advantages, challenges remain in the clinical translation of hydrogel-based drug delivery systems. Key issues include optimizing the mechanical strength, degradation rate, and biocompatibility of hydrogels for specific applications, as well as ensuring reproducibility and scalability for large-scale production [221]. Furthermore, the long-term stability and potential immune response to hydrogel materials need to be thoroughly investigated to ensure safety and efficacy. Future research is focused on developing multifunctional hydrogels that combine drug delivery with diagnostic capabilities, enabling theragnostic applications. The field is increasingly leveraging advanced fabrication methods, including 3D and 4D printing, to engineer adaptive drug delivery platforms that can dynamically respond to physiological cues [222]. Furthermore, incorporating artificial intelligence (AI) and machine learning (ML) into hydrogel design could significantly improve their performance and broaden their applicability.

## 5. Printing Technologies and Material Requirements

In 4D printing, the fourth dimension—time—is imparted not by the printing process alone, but by the controlled application of external stimuli to pre-programmed printed architectures. After fabrication, stimuli such as temperature, moisture, pH, light, electric fields, or magnetic fields are applied globally or locally to activate shape or property changes. The printed geometry, material distribution, and network anisotropy determine how the structure responds to the applied stimulus, enabling predictable and programmable deformation over time [223]. This capability enables reversible, sequential, or multi-stage transformations, thereby transforming hydrogels from passive materials into dynamic, programmable systems with adaptive and reconfigurable behavior that cannot be achieved using static 3D-printed constructs [38,40].

**Figure 17:**
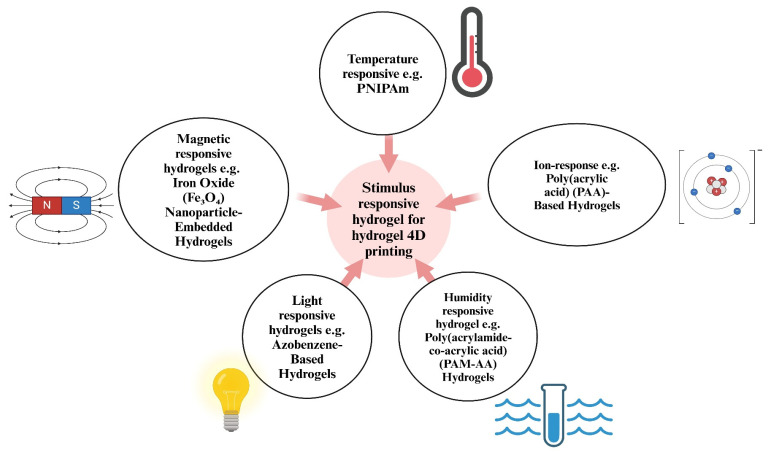
Summary of stimuli-responsive hydrogel materials for developing 4D—printed hydrogels.

Most AM processes can accommodate 4D printing, provided the printed material, or precursor material, is compatible with the printer [224,225]. Among the AM methods discussed, material extrusion techniques (e.g., direct ink writing) and vat photopolymerization approaches (e.g., Stereolithography (SLA) and Digital Light Processing (DLP)) are the most widely adopted for hydrogel-based 4D printing, although they offer distinct advantages and limitations. Material extrusion methods provide broad material compatibility, facile rheological tuning, and straightforward implementation of multilayer, gradient, and anisotropic architectures, making them particularly suitable for large, soft, and highly deformable hydrogel actuators and soft robotic systems [131,226,227,228,229,230,231,232,233,234,235,236,237,238]. However, these methods are generally limited in printing resolution. In contrast, vat photopolymerization enables higher spatial resolution and faster fabrication, allowing precise control over complex geometries and micro-scale features, but it requires photo-crosslinkable formulations and can restrict material diversity and multi-material integration [129,133,159]. Other AM methods, including material jetting, binder jetting, powder bed fusion, and directed energy deposition, remain far less compatible with hydrogel printing due to viscosity constraints, post-processing requirements, or high-energy inputs that are unsuitable for water-rich, stimuli-responsive materials [13,19]. Various printing techniques, including inkjet, extrusion, and stereolithography, present unique advantages and challenges in the production of hydrogels. Table 2. shows the classification of AM processes according to ISO/ASTM 52900: brief descriptions, raw material forms, advantages, and disadvantages. Among the seven categories of AM technologies, material extrusion and vat photopolymerization have made extensive use of polymeric materials [229]. Figure 17 presents a summary of stimuli-responsive hydrogel materials for developing 4D-printed hydrogels.

### 5.1. Mechanical Properties and Structural Design of Stimuli-Responsive Hydrogels for 4D Printing

The integration of stimuli-responsive hydrogels into 4D printing necessitates precise engineering of their mechanical properties—including tensile strength, elasticity, toughness, and fatigue resistance—to ensure both printability and functional performance under dynamic conditions. A recent study emphasizes that the success of 4D-printed polymeric and hydrogel systems depends not only on stimulus responsiveness but also on the ability to dynamically tune mechanical properties through structural design and material programming, enabling time-dependent shape transformation and functional adaptability [230]. These advancements have leveraged multi-network architectures, which have enabled materials that withstand repeated actuation cycles without fracture. For instance, ionically–covalently crosslinked PEGDA/chitosan hydrogels fabricated via extrusion-based 3D printing have demonstrated elastic moduli up to ~3.8 MPa, tensile strengths exceeding 7 MPa, large extensibility (>160%), fracture energies on the order of 10^3^ J·m^−2^, and excellent fatigue resistance under cyclic loading while maintaining robust printability due to shear-thinning and yield-stress behavior, making them suitable for mechanically demanding and multifunctional soft systems [231]. Similarly, gelatin-based hydrogels constructed from dynamic covalent imine/Diels–Alder double networks exhibit high stretchability (elongation at break >500%), recoverable mechanical integrity via self-healing, and stable load-bearing capability during repeated deformation, supporting their use in mechanically adaptive 4D-printed structures [232]. Nanocomposite reinforcement strategies further enhance mechanical robustness and functional integration; for example, nanocellulose-reinforced PNIPAM-based bilayer hydrogels exhibit improved modulus, toughness, and interfacial strength for thermally driven actuation and self-sensing, while MXene-based conductive organohydrogels achieve ultrahigh stretchability (up to ~2000%), high toughness, and stable electromechanical performance under large cyclic strains, extending the mechanical design space for adaptive and multifunctional hydrogel systems [233,234].

Achieving stimuli-triggered mechanical adaptability—where properties such as stiffness or swelling ratio change on-demand—is central to 4D printing applications. Thermo-responsive hydrogels like PNIPAm exhibit a sharp modulus transition near their lower LCST, enabling temperature-driven shape morphing and actuation [39]. Light-responsive systems incorporating photochromic molecules, particularly azobenzene derivatives, undergo reversible Trans–Cis Isomerization (E–Z photoisomerization) under light irradiation, leading to controllable stiffening/softening, reversible adhesion, and spatially programmable mechanical patterning within hydrogel networks [235]. Recent work has also explored piezoelectric and triboelectric additives—such as BaTiO_3_ ceramics, ZnO nanostructures, and polymer–ceramic composites—to create mechano-responsive hydrogel-based systems capable of self-sensing, energy harvesting, and self-powered operation under cyclic deformation [38]. Moreover, the emergence of machine learning-assisted design and multi-material printing now allows for the fabrication of hydrogel systems with graded or anisotropic mechanical properties that mimic biological tissues, opening new avenues in tissue engineering, wearable bioelectronics, and adaptive soft robotics [38,40]. Future efforts should focus on establishing predictive models that link molecular design, printing parameters, and post-printing mechanical behavior, thereby accelerating the development of robust, stimuli-responsive hydrogel platforms for next-generation smart devices.

**Table 2:** Classification of AM processes according to ISO/ASTM 52900: brief descriptions, raw material forms, advantages, and disadvantages.

AM Process Classification	Brief Description	Examples of Technique	Form of Raw Material	Hydrogel Compatibility	Pros	Cons	References
Binder Jetting	A liquid bonding agent is selectively applied to a thin layer of powder spread across a powder bed, effectively binding the powder particles in specified areas.	Metal binder jetting, sand binder jetting, ceramic binder Jetting.	Ceramics, metal, biomaterials, polymers	Low	High build rate, incorporating functionally graded materials.	Resolution and accuracy, post-processing	[236,237,238]
Directed Energy Deposition	Focused thermal energy is employed to melt materials during deposition, facilitating their fusion. The source of thermal energy can be a laser, electron beam, or plasma arc.	Electron beam AM, laser engineering net shaping	Metals, alloys, and composites	Not Suitable	High-throughput new material development, rapid manufacturing of large near-net-shape parts.	Shrinkage, residual stress, and deformation	[236,237,239]
Material jetting	Droplets of build materials, such as fine inorganic powders suspended in organic solvents with photopolymer or waxes, are selectively deposited to construct the desired part.	PolyJet, NanoParticle Jetting	Polymers	Moderate	High resolution and accuracy, multiple materials and colors, smooth surface finishes	Limited material selection, strength	[236,237,240]
Material Extrusion	Material is selectively dispensed through a heated nozzle using a powder injection molding feedstock, which consists of metal powder combined with organic binders and formed into coils, rods, or granules. This material is extruded through the heated nozzle and deposited onto a platform that has precise x, y, and z motion control to create the desired geometric shape. Afterward, the part undergoes debinding and consolidation processes to achieve the final product.	Direct ink writing, fused filament fabrication	Polymers	High (Preferred)	Low costs, a large variety of feedstock materials	Impaired surface quality, part deformation	[236,237,241]
Powder Bed Fusion	Thermal energy is employed to selectively fuse specific regions of a powder bed, defining the final part geometry. The energy source, which may be a laser or an electron beam, either sinters or melts the powders depending on the material and the intensity of the applied energy.	Selective laser sintering, direct metal laser sintering, electron beam melting, selective heat sintering	Metals, polymers	Not Suitable	Homogeneous microstructures, free of internal stresses	High cost, rough surface finish	[236,237,242]
Sheet Lamination	Thin sheets or foils of material are shaped using a laser or knife and then bonded together to create a 3D part. This process does not require sintering.	Laminated object manufacturing, ultrasonic AM	Metals, polymers	Low	Hybrid manufacturing integration, ease of material handling	Surface finish, limited material selection	[236,237,243]
Vat Photo- polymerization	A liquid photopolymer mixed with metal or ceramic powder is selectively hardened using light-activated polymerization. When metals or ceramics are involved, the polymer breaks down, and the shape is solidified through a sintering process.	Scanning stereolithography, microstereolithography, two-photon polymerization	Polymers	High (Preferred)	High precision, smooth finish, versatile material options	Limited material strength, limited build volume	[236,237,244]

### 5.2. Extrusion-Based Printing Techniques

Extrusion printing is a method that builds structures by extruding material through a nozzle onto a platform. The material can be manipulated either by moving the nozzle across the platform or by shifting the platform beneath the nozzle. In both scenarios, 3D structures are created by continuously depositing material layer by layer. However, extrusion printing encounters several challenges, including difficulties with polymerization kinetics. If the kinetics are too slow, it can become challenging to maintain the dimensional stability of the printed form.

Among extrusion-based additive manufacturing techniques, direct ink writing (DIW) and fused filament fabrication (FFF) are commonly discussed in the context of hydrogel processing. DIW is predominantly used for soft, shear-thinning, water-rich hydrogels, whereas FFF is primarily applied to thermoplastic-based or composite filaments and is therefore less suitable for conventional hydrogel formulations [245]. As a result, DIW remains the most widely used and versatile technique for hydrogel-based 4D printing in the literature. Accordingly, this section focuses on the DIW technique, its capabilities, limitations, and representative recent studies.

#### Direct Ink Writing

DIW is a widely used extrusion-based AM process, offering excellent interfacial bonding and adjustable mechanical properties [19]. In this process, viscoelastic ink is extruded through a deposition nozzle in a layer-by-layer manner to create scaffolds and other 3D geometries on a computer-controlled translational stage [226,246]. DIW distinguishes itself from other additive manufacturing technologies because it is not limited by the material class, as long as the precursor ink possesses suitable rheological properties, including appropriate viscosity, yield stress under shear and compression, and viscoelastic characteristics such as loss and elastic moduli. As a result, this technique can effectively print nearly any ink into a 3D structure with high-resolution patterning, architectural flexibility, and specific material properties [247].

Hydrogels do not always need to be modified for DIW fabrication; instead, they can be used to enhance the properties of other materials. This versatility opens up exciting possibilities for innovative applications and cutting-edge solutions. For instance, Shiyu Qin et al. [248] modified the properties of all-aromatic polyimides by adding polyamide acid salt-based hydrogels. Polyacrylic Acid Sodium Salt (PAAS) hydrogels exhibit shear-thinning behavior, sufficient yield stress, and rapid structural recovery, which are critical rheological characteristics for DIW. These properties enable smooth extrusion through the nozzle while maintaining shape fidelity and layer stability after deposition. DIW was employed to fabricate PI objects, resulting in a reduction of thermal conductivity from 0.102 W/m·K to 0.061 W/m·K after structural design and 3D printing. Furthermore, the density decreased from 0.4562 g/cm^3^ to 0.2731 g/cm^3^.

**Figure 18:**
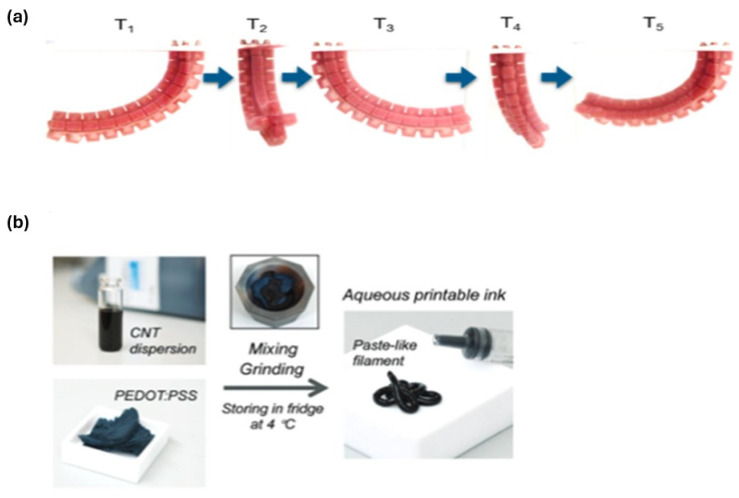
(**a**) The motion of the artificial tentacle over time from T1 to T5, showcasing a complete circular rotation [249]. (**b**) Process for preparing composite inks using DIW [250].

In an article related to the alteration of rheological properties of hydrogels, Yin Cheng et al. [249] utilized biocompatible alginate as a rheological modifier to develop 3D freeform architectures of both chemically and physically cross-linked hydrogels using DIW printing. To examine the effects of rheological tailoring, researchers chose acrylamide as the model hydrogel precursor and incorporated varying amounts of alginate. It should be noted that in this work, alginate is employed solely as a rheological modifier to enable direct ink writing and does not contribute to stimulus-responsive actuation. Meanwhile, the stimuli-responsive behavior originates from the host hydrogels, including hydraulic actuation in PAM systems, pneumatic deformation in PVA structures, and thermo-/photothermal response in PNIPAM-based actuators. Figure 18a illustrates the prepared artificial tentacle structure rotation at different time intervals.

Ji Liu et al. [250] used the DIW 3D printing technique to manufacture multifunctional conducive polymer composite hydrogels. In this study, a highly 3D-printable ink was created using PEDOT, relying solely on commercially available raw materials. To achieve the desired properties, CNTs were added in the printable ink. This provides effective electromagnetic interference shielding and exhibits strong sensing capabilities. Moreover, its biocompatibility highlights its significant potential for use in implantable devices and tissue engineering applications. Figure 18b demonstrates the preparation process for the ink.

The ink should possess shear thinning properties, meaning it flows through the deposition nozzle like a liquid when stressed above its yield strength and quickly sets after deposition to ensure shape retention. Since shear thinning is not an inherent property for most inks, especially dilute ones, many inks are excluded or require meticulous control of their compositions and rheological behaviors [251].

In another article [252], hybrid scaffolds were developed by combining either alginate or alginate-bio glass composite hydrogels with a 3D-printed porous PLA structure. The deposited PLA scaffolds underwent surface treatment with polyacrylic acid, significantly enhancing their wettability. This surface-modified PLA scaffold integrated seamlessly with the hydrogels, offering both shape and mechanical rigidity to the hydrogel structure.

Natural hydrogels are inherently weak, causing the printed filaments to spread easily. If these filaments fail to retain their shapes, it negatively affects the subsequent layers and the overall structure, as the initial layers may collapse or deform under the weight of the layers above them. Consequently, stacking a natural hydrogel into a 3D construct is highly challenging [253].

4D printing of hydrogels represents a promising frontier in additive manufacturing, enabling the fabrication of dynamic, stimuli-responsive structures with applications in biomedical engineering, soft robotics, and smart materials [254]. By leveraging various AM techniques, such as vat photopolymerization, extrusion-based printing, and material jetting, researchers have developed hydrogels with tunable mechanical properties, self-healing abilities, and complex architectures. However, significant challenges remain in optimizing print resolution, improving interlayer adhesion, and enhancing the mechanical robustness of hydrogel-based structures. The high water content in hydrogels often compromises structural stability, particularly in stereolithography-based printing, limiting the fabrication of intricate geometries. Additionally, extrusion-based techniques like DIW require precise rheological control to maintain print fidelity and prevent filament collapse. Further advancements in multi-material printing, hybrid hydrogel formulations, and post-processing strategies are essential to overcome these limitations [255,256].

### 5.3. Vat Photopolymerization

The primary limitations of 3D printing techniques are their structural resolution and the requirement for inks to possess specific physical properties, imposing constraints on ink composition. However, certain challenges, particularly the resolution issue, can be overcome by utilizing stereolithography-based printing systems [257]. Vat photopolymerization usually produces highly crosslinked polymer networks [258]. Among the various vat photopolymerization techniques, SLA stands out as it utilizes a laser to create a focused beam [259], and DLP uses a projector to illuminate a specific area [86] in the manufacturing of 4D hydrogels.

#### 5.3.1. Stereolithography

SLA is a vat polymerization technique that involves layers of liquid precursor, which consist of a monomer, a crosslinker, a PI, and a photoabsorber or a pre-polymer [86,260,261] in a vat are sequentially exposed to UV light, selectively solidifying them. A PI molecule in the resin reacts to incoming light, activating the chemical polymerization reaction locally and curing only the exposed regions. It utilizes photopolymerizable materials, such as acrylates and methacrylamides, as the polymer resin. These materials are cured by a light source, enabling the construction of the desired 3D structures in a layer-by-layer manner [262]. Once the first layer is formed, a new layer of resin is applied, irradiated, and cured. As a result, the part gradually grows incrementally, layer by layer [102]. Controlling the light intensity and exposure duration enables precise local control over monomer conversion and crosslinking density, which affects local swelling [100].

Ilbey Karakurt et al. [263] prepared ascorbic acid-loaded hydrogels using bottom-up Stereolithography (SLA). The hydrogel is composed of Acrylic Acid (AA) encapsulated within a Poly(ethylene glycol) Diacrylate (PEGDMA)-based polymer network, polymerized using riboflavin as a Photoinitiator (PI). Arfa S. Alketbi et al. [264] studied the influence of PEGDA photopolymerization on 3D-printed hydrogel structure and swelling in micro-stereolithography. The cross-linking reaction of PEGDA was conducted under light exposure, using Diphenyl(2,4,6-trimethylbenzoyl)phosphine Oxide (TPO) as the PI, which produces free radicals. To illustrate the impact of exposure energy, PEGDA gels were prepared at intensities of 20 and 30, corresponding to 43.3 mW/cm^2^ and 60 mW/cm^2^, respectively, with exposure durations of 1, 2, and 2.5 s. In another study related to the photopolymerization of hydrogels, Manjot Singh et al. [265] investigated the photopolymerization of hydrogels with closed-loop control involved preparing PEGDMA precursor solutions at concentrations of 8%, 10%, and 12% weight/volume by dissolving PEGDMA in deionized water. Similarly, NIPAm precursor solutions were prepared at the same concentrations by dissolving 0.5 g of NIPAm and 0.5 g of 1% MBA in DI water, with the total volume adjusted using 5.25 g, 4 g, or 3.17 g of DI water to achieve the desired concentrations. A PI solution containing 0.2% of 20% 2,2-dimethoxy2-phenylacetophenone in ethanol was used. The researchers discovered that closed-loop photopolymerization allowed for the fabrication of hydrogels with a controlled network structure and storage modulus. They also observed that the network structure and storage modulus of PEGDMA hydrogels depended on the extent and temporal profile of UV light exposure.

The SLA technique has achieved only limited success in printing hydrogels that possess intricate and complex internal structures. This is attributed to the high water content relative to the reactive monomers, which compromises the layer-by-layer build due to insufficient adhesion on the build plate. Additionally, it is challenging to form a structure that can support itself against gravity [266].

#### 5.3.2. Digital Light Processing

**Figure 19:**
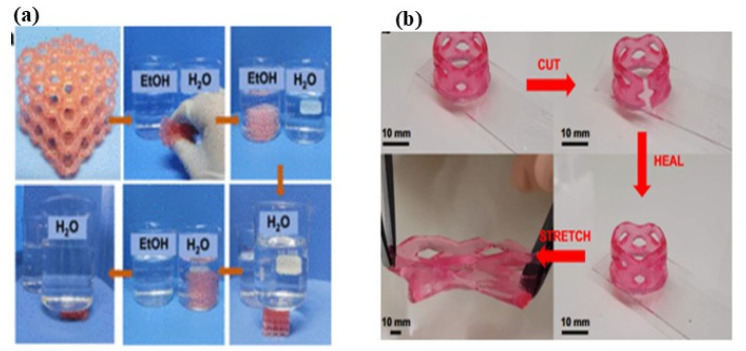
(**a**) The printed soft lattice structure transitioned to a rigid state after being immersed in ethanol for 10 min and reverted to its original soft state upon immersion in distilled water for 2 min [268]. (**b**) A holed cylindrical structure printed with methyl red sodium salt dye demonstrated the ability to withstand stretching deformation after a 2 h healing process [270].

DLP technology, named for the digital light projector, employs digital micro-mirror device technology [267]. In this process, photosensitive resin is locally polymerized to form a stack of layers through consecutive projections of 2D layer images from a DLP source. Yangyang He et al. [268] prepared the transparent, strong, and highly conductive hydrogels using the DLP 4D printing technique. A novel waterborne polyurethane microemulsion is synthesized utilizing a standard hydrophobic PI. The high efficacy of this PI enables its application in DLP 3D printing, allowing the creation of intricate structures with high resolution. Figure 19a illustrates the transformation of a printed lattice for ethanol-induced rigidity and water recovery.

Zhengqiang Guo et al. [269] prepared cellulose with high toughness and strength by using a DLP 3D printing strategy for strain sensing. The tough hydrogels were prepared using a dual-crosslinking strategy that combines both physical and chemical methods. First, CNF was dispersed in deionized water. Then, sodium dodecyl sulfate and sodium chloride were added, and the solution was mixed with a magnetic stirrer to achieve a uniform dispersion. PEGDA was added as a crosslinker and stirred thoroughly. Researchers state that hydrogel possesses excellent self-powered characteristics and broad environmental adaptability, making it highly advantageous for the future development of wearable electronic devices. Matteo Caprioli et al. [270] developed self-healing hydrogels through 3D printing using DLP. Water-soluble nanoparticles based on diphenyl (2,4,6-trimethylbenzoyl) phosphine oxide (containing ionic surfactant methyl red sodium salt) were employed as a PI. Figure 19b represents the hole cylinder structure.

The potential of DLP-based 3D printing to fabricate hybrid structures combining highly stretchable, high-water-content hydrogels with other UV-curable polymers has yet to be realized. This is primarily due to the limited availability of highly efficient DLP-based multimaterial 3D printing systems and the absence of a universal method for creating robust bonds between high-performance hydrogels and various UV-curable polymers [271,272,273].

### 5.4. Role of Rheology in Additive Manufacturing and 4D Printing

Rheological properties are fundamental determinants of success in the AM of hydrogels and are critical for reliable 4D printing. These properties govern key process phenomena, including filament formation, extrusion fidelity, shape retention, and interlayer adhesion, thereby dictating the structural and functional outcomes of the printed construct [13,226]. While stimuli-responsive chemistry controls post-printing shape or property transformation, rheology determines whether complex, programmable architectures can be fabricated without collapse or distortion [274]. For extrusion-based additive manufacturing techniques, such as DIW, suitable rheological behavior necessitates a precise balance of key viscoelastic properties. Specifically, the ink must exhibit pronounced shear-thinning viscosity to enable smooth extrusion under applied shear, a finite yield stress to prevent uncontrolled flow post-deposition, and rapid structural recovery to ensure shape fidelity and interlayer cohesion [226,275]. Ensuring shear-thinning behavior is a critical rheological requirement for hydrogel inks, as it enables smooth extrusion through the nozzle under shear while allowing rapid viscosity recovery after deposition, thereby preserving filament shape fidelity and print resolution. Hydrogels that lack this balance may exhibit excellent stimuli responsiveness but fail during printing, demonstrating that printability and functional responsiveness are not intrinsically coupled [222].

In 4D printing, rheology governs not only manufacturability but also the programmed time-dependent deformation of the printed structure. By spatially tuning viscoelastic properties, it is possible to engineer multilayer, gradient, and anisotropic architectures that prescribe specific deformation directions, curvatures, and actuation sequences in response to external stimuli [158]. Many high-performing 4D-printed hydrogel systems achieve reliable actuation not solely due to enhanced stimuli sensitivity, but through deliberate rheological engineering. This is often accomplished by incorporating fillers, secondary polymer networks, or supramolecular interactions, which serve to significantly enhance the ink’s yield stress and elastic recovery properties [172].

## 6. Challenges and Future Opportunities

**Figure 20:**
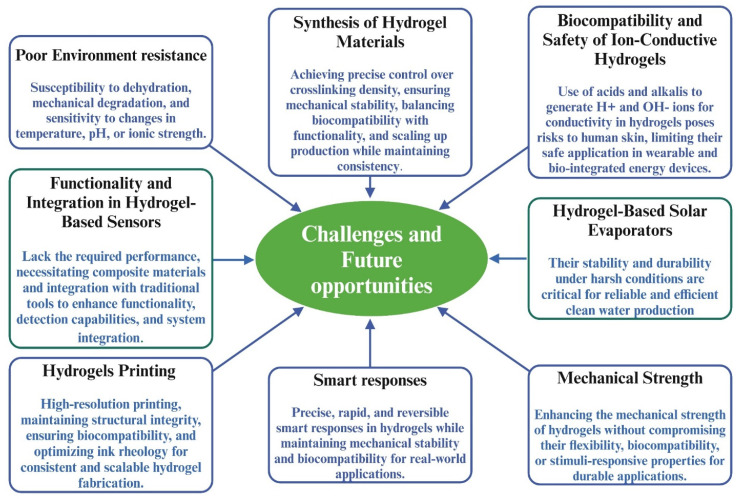
Challenges and opportunities in hydrogels.

Recently, a wide variety of innovative hydrogels have been reported in the literature, inspired by the structural and functional principles governing behavior in the natural world. Figure 20 illustrates the opportunities and challenges in hydrogel research. Many of these approaches to designing multifunctional networks offer forward-thinking concepts for the new structural design of advanced materials [276]. Hydrogels have achieved remarkable progress; however, there are significant challenges in terms of their fabrication, mechanical properties, and compatibility with required applications.

(1) For instance, conventional conductive hydrogels are limited to moderate environments due to their poor environmental resilience. Their high water content and hydrophilic structure cause them to swell undesirably in humid conditions, freeze at sub-zero temperatures, and dehydrate through evaporation, compromising their structural integrity [277]. However, conductive hydrogels are typically designed to achieve desired properties by incorporating many conductive fillers. However, this often weakens the gels’ mechanical properties due to reduced network compatibility caused by the aggregation of conductive materials. As a result, this restricts their practical applications, particularly in wearable electronics, which necessitate a blend of high conductivity, stretchability, fracture strength, appropriate modulus, and quick self-recovery. Therefore, ongoing efforts are necessary to meticulously choose the raw materials, optimize fabrication methods, and refine structural designs [278]. Moreover, developing suitable ink for printing requires balancing tunable rheology with printing quality, as highly concentrated ink offers rapid prototyping. Still, it can result in rigid, less-responsive devices, while dilute ink improves flexibility but sacrifices shape fidelity and speed [279].
(2) Natural hydrogels derived from renewable and cost-effective sources like starch form a fascinating category of biopolymeric materials. They are being progressively employed in a diverse range of applications spanning the biomedical, cosmeceutical, and food industries. However, the synthesis of these materials is hampered by lengthy processing times, high energy consumption, and safety concerns, which often result in significant environmental damage. These issues are major obstacles to their broader utilization [280]. There are several limitations related to the printability of natural hydrogels; for example, the mechanical performance, specifically the elastic modulus, is lower in the permanent state compared to the temporary state after the printing [281].
(3) Hydrogels have emerged as promising materials for energy conversion and storage systems. Most ion-conductive hydrogel electrolytes derive their conductivity from the movement of H^+^ and OH^-^ ions. However, generating these ions typically requires the use of acids and alkalis, which can be harmful to human skin. This presents a challenge for their application in wearable electronic devices. While the preparation methods and technologies for hydrogel electrolytes are relatively mature, there is significant room for improvement in material selection. Developing environmentally and socially sustainable materials, or using biodegradable hydrogels as electrolyte or electrode materials, is an urgent need [282].
(4) Another area where hydrogels are making strides is sensors. Hydrogel material alone often falls short of meeting application demands, so composite materials are used to introduce additional functionalities. The synergy between hydrogels and other traditional analytical tools is leveraged. While hydrogels do not always surpass existing techniques, they enhance functionality, facilitate detection, and integrate multiple sensor components into a single system.
(5) Hydrogel-based evaporators have outperformed many reported evaporators, offering distinct advantages. However, challenges and opportunities remain to enhance their strengths. For instance, a deeper understanding of the fundamental evaporation mechanism within hydrogels is needed. Additionally, the stability and durability of hydrogel-based solar evaporators under severe conditions require improvement to ensure stable clean water delivery in practical applications [283].
(6) Achieving high-strength hydrogels requires overcoming limitations such as balancing mechanical robustness with flexibility, ensuring biocompatibility, and enhancing stability under varying environmental conditions. Developing tunable mechanical properties for specific applications, integrating multiple enhancement strategies for synergistic effects, and understanding structure–property relationships through advanced characterization techniques remain critical hurdles [284,285]. Additionally, creating scalable and sustainable fabrication methods while maintaining performance consistency poses significant challenges.
(7) The widespread adoption of 3D hydrogel printing faces several critical hurdles, including optimizing material properties for improved printability and bioactivity, enhancing printing techniques for higher resolution and speed, and designing complex scaffold architectures with functional gradients and vascular networks. Post-printing processing, such as ensuring structural integrity and functionality, along with the challenges of scaling up to high-throughput manufacturing, further complicate its implementation. Additionally, the clinical translation of 3D-printed hydrogels requires addressing regulatory and ethical considerations, as well as ensuring reproducibility and biocompatibility for real-world applications [286].

Hydrogels have achieved tremendous development in terms of their range of applications. Their chemical diversity and control over multiscale architecture have made them attractive in the fields of medicine, soft robotics, sensors, and environmental engineering. However, there are significant challenges related to their printing, mechanical properties, biocompatibility, and cost-effectiveness. The rapid advancements in hydrogel technology present exciting opportunities across diverse fields, driven by their unique properties and multifunctionality. In wearable electronics, the development of conductive hydrogels with enhanced environmental resilience, mechanical strength, and self-recovery capabilities can revolutionize flexible and stretchable devices. For biomedical applications, natural hydrogels derived from renewable sources offer sustainable solutions, with potential improvements in synthesis methods to reduce energy consumption and environmental impact. In energy storage and conversion, the creation of biocompatible and biodegradable hydrogel electrolytes can address safety concerns and expand their use in wearable and eco-friendly devices. Hydrogel-based sensors provide opportunities for integrating multiple functionalities into compact systems, enhancing detection and monitoring capabilities. Additionally, hydrogel evaporators hold promise for efficient clean water production, with further research into evaporation mechanisms and durability under harsh conditions. The pursuit of high-strength hydrogels with tunable mechanical properties opens doors for applications in tissue engineering, soft robotics, and beyond. Finally, hydrogel printing offers transformative potential in regenerative medicine, with opportunities to optimize printing techniques, scaffold designs, and clinical translation processes. By addressing current challenges and leveraging interdisciplinary innovations, hydrogels can unlock groundbreaking solutions to global challenges in healthcare, sustainability, and technology.

## 7. Conclusions

This review has provided a comprehensive and integrative examination of stimuli-responsive hydrogels, encompassing their classification by source, structure, and crosslinking strategies, their behavior under a wide spectrum of external stimuli, and the transformative role of 4D printing in enabling programmable, shape-morphing systems. By synthesizing developments in hydrogel chemistry, smart material design, and advanced manufacturing, we proposed a multi-dimensional framework linking stimulus types to material compositions, fabrication methods, and application domains.

Stimuli-responsive hydrogels continue to unlock opportunities across a range of engineering and scientific fields—from biomedical devices and soft robotics to water purification and smart sensors. Yet, persistent limitations such as weak mechanical strength, slow actuation speeds, and challenges in printability and scalability remain critical bottlenecks. Addressing these requires collaborative efforts across chemical engineering, materials science, and bio-fabrication to develop robust, multifunctional, and industrially viable systems.

Looking ahead, the convergence of intelligent material systems with emerging digital manufacturing platforms will be central to realizing hydrogels as core components of next-generation engineering technologies. By bridging material function with practical application, stimuli-responsive hydrogels are poised to play a pivotal role in shaping the future of adaptive, sustainable, and high-performance systems in chemical and process engineering.

## Data Availability

No new data were created or analyzed in this study.

## List of Abbreviation

**3DP**	Three-Dimensional Printing
**4D Printing**	Four-Dimensional Printing (3D printing with time-dependent transformation)
**AA**	Acrylic Acid
**AAC**	Acrylic Acid Network
**AFM**	Atomic Force Microscopy
**AL**	Alginate
**ALP**	Alkaline Phosphatase
**AM**	Additive Manufacturing
**APS**	Ammonium Persulfate
**ARS**	Alizarin Red S
**ASTM**	American Society for Testing and Materials
**BA**	Benzyl Acrylate
**BGC**	Bioactive Glass Ceramic
**BMP**	Bone Morphogenetic Protein
**BMP-2**	Bone Morphogenetic Protein-2
**BTE**	Bone Tissue Engineering
**Ca^2+^**	Calcium Ion
**CAD**	Computer-Aided Design
**Ca-P**	Calcium Phosphate
**CMC**	Carboxymethyl Cellulose
**CNF**	Cellulose Nanofiber
**CNT**	Carbon Nanotube
**CS**	Chitosan
**CSG**	Chitosan-Based Hydrogel
**CTE**	Cartilage Tissue Engineering
**DED**	Directed Energy Deposition
**DIW**	Direct Ink Writing
**DLW**	Direct Laser Writing
**DLP**	Digital Light Processing
**DMAPMA**	**N**,N-Dimethylaminopropyl Methacrylamide
**DN**	Double Network
**EBE**	Electron Beam Energy
**EBM**	Electron Beam Melting
**ECM**	Extracellular Matrix
**EDGMA**	Ethylene Glycol Dimethacrylate
**EO**	Ethylene Oxide
**E–Z**	Trans–Cis Isomerization
**FFF**	Fused Filament Fabrication
**GAG**	Glycosaminoglycan
**GelMA**	Gelatin Methacryloyl
**GO**	Graphene Oxide
**HA**	Hydroxyapatite
**HEMA**	2-Hydroxyethyl Methacrylate
**IPN**	Interpenetrating Polymer Network
**ISO**	International Organization for Standardization
**LCST**	Lower Critical Solution Temperature
**LAP**	Lithium Phenyl-2,4,6-Trimethylbenzoylphosphinate
**LOM**	Laminated Object Manufacturing
**MBA–N**	N′-Methylenebisacrylamide
**MC**	Methylcellulose
**MMP**	Matrix Metalloproteinase
**MNP**	Magnetic Nanoparticle
**MXene**	Two-Dimensional Transition Metal Carbide/Nitride
**Mw**	Molecular Weight
**NBG**	Nano-Bioglass
**NIPAM**	N-Isopropylacrylamide
**NIR**	Near-Infrared
**OEGMA**	Oligo(ethylene glycol) Methacrylate
**OCN**	Osteocalcin
**PAA**	Poly(acrylic acid)
**PAAm**	Polyacrylamide
**PAM**	Polyacrylamide
**PANI**	Polyaniline
**PBS**	Phosphate-Buffered Saline
**PEC**	Polyelectrolyte Complex
**PEG**	Poly(ethylene glycol)
**PEGDA**	Poly(ethylene glycol) Diacrylate
**PEDOT:PSS**	Poly(3,4-ethylenedioxythiophene):Poly(styrenesulfonate)
**PET**	Poly(ethylene terephthalate)
**PHBV**	Poly(3-hydroxybutyrate-co-3-hydroxyvalerate)
**PI**	Photoinitiator
**PLA**	Polylactic Acid
**PLGA**	Poly(lactic-co-glycolic acid)
**PLLA**	Poly(L-lactic acid)
**PNIPAM**	Poly(N-isopropylacrylamide)
**PNVCL**	Poly(N-vinylcaprolactam)
**PSL**	Projection-Based Stereolithography
**RGO**	Reduced Graphene Oxide
**RH**	Relative Humidity
**SCF**	Short Carbon Fiber
**SEM**	Scanning Electron Microscopy
**SFF**	Solid Freeform Fabrication
**SLA**	Stereolithography
**SLS**	Selective Laser Sintering
**SLM**	Selective Laser Melting
**SPION**	Superparamagnetic Iron Oxide Nanoparticle
**TAG**	Tannic Acid–Gelatin
**TE**	Tissue Engineering
**TEMED**	N,N,N′,N′-Tetramethylethylenediamine
**TGF-β**	Transforming Growth Factor-Beta
**TOCN**	TEMPO-Oxidized Cellulose Nanofiber
**TPO**	Diphenyl(2,4,6-trimethylbenzoyl)phosphine Oxide
**UV**	Ultraviolet
**VAT**	Vat Photopolymerization
**WD**	Wound Dressing
**WMR**	Walking Magnetic Robot
